# Larval body patterning and apical organs are conserved in animal evolution

**DOI:** 10.1186/1741-7007-12-7

**Published:** 2014-01-29

**Authors:** Heather Marlow, Maria Antonietta Tosches, Raju Tomer, Patrick R Steinmetz, Antonella Lauri, Tomas Larsson, Detlev Arendt

**Affiliations:** 1 European Molecular Biology Laboratory, Development Biology Unit, EMBL Heidelberg, Meyerhofstraße 1, 69117 Heidelberg, Germany; 2 Present address: Howard Hughes Medical Institute, Stanford University, Stanford, CA 94305, USA; 3 Present address: Department for Molecular Evolution and Development, Centre for Organismal Systems Biology, University of Vienna, Althanstraße 14, A-1090 Vienna, Austria; 4 Present address: Institute for Biological and Medical Imaging, Helmholtz Zentrum München, Ingolstädter Landstr. 1, 85764 München, Germany; 5 European Molecular Biology Laboratory, Structural and Computational Biology Unit, Meyerhofstraße 1, 69117 Heidelberg, Germany

**Keywords:** Apical-blastoporal axis, Apical organ, Body plan, Larval evolution

## Abstract

**Background:**

Planktonic ciliated larvae are characteristic for the life cycle of marine invertebrates. Their most prominent feature is the apical organ harboring sensory cells and neurons of largely undetermined function. An elucidation of the relationships between various forms of primary larvae and apical organs is key to understanding the evolution of animal life cycles. These relationships have remained enigmatic due to the scarcity of comparative molecular data.

**Results:**

To compare apical organs and larval body patterning, we have studied regionalization of the episphere, the upper hemisphere of the trochophore larva of the marine annelid *Platynereis dumerilii*. We examined the spatial distribution of transcription factors and of Wnt signaling components previously implicated in anterior neural development. Pharmacological activation of Wnt signaling with Gsk3β antagonists abolishes expression of apical markers, consistent with a repressive role of Wnt signaling in the specification of apical tissue. We refer to this Wnt-sensitive, *six3*- and *foxq2*-expressing part of the episphere as the ‘apical plate’. We also unraveled a molecular signature of the apical organ - devoid of *six3* but expressing *foxj*, *irx*, *nkx3* and *hox* - that is shared with other marine phyla including cnidarians. Finally, we characterized the cell types that form part of the apical organ by profiling by image registration, which allows parallel expression profiling of multiple cells. Besides the *hox*-expressing apical tuft cells, this revealed the presence of putative light- and mechanosensory as well as multiple peptidergic cell types that we compared to apical organ cell types of other animal phyla.

**Conclusions:**

The similar formation of a *six3+, foxq2+* apical plate, sensitive to Wnt activity and with an apical tuft in its *six3-*free center, is most parsimoniously explained by evolutionary conservation. We propose that a simple apical organ - comprising an apical tuft and a basal plexus innervated by sensory-neurosecretory apical plate cells - was present in the last common ancestors of cnidarians and bilaterians. One of its ancient functions would have been the control of metamorphosis. Various types of apical plate cells would then have subsequently been added to the apical organ in the divergent bilaterian lineages. Our findings support an ancient and common origin of primary ciliated larvae.

## Background

**Figure 1:**
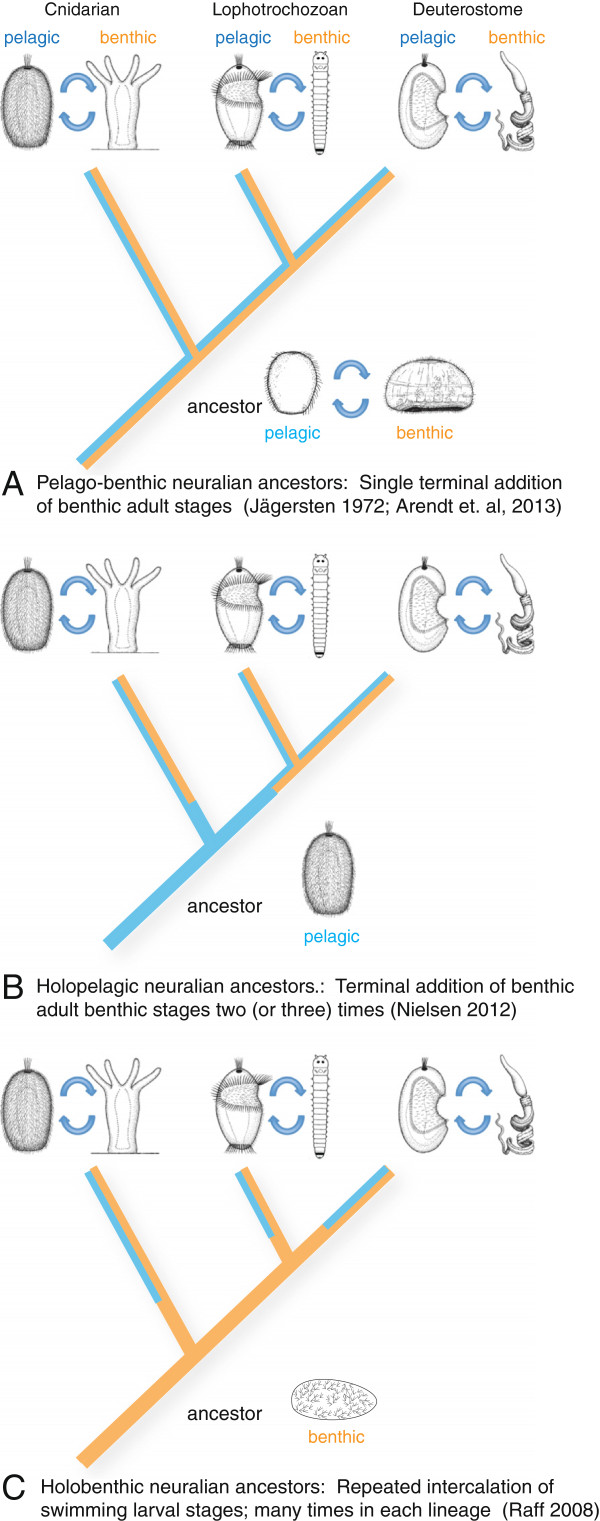
**The three scenarios show the origin of the pelagic larval body plan, indicated by red arrows.** The presence of pelagic forms is indicated by blue lines and that of benthic forms by brown lines. A single-coloured line indicates a monophasic life cycle that would be pelagic in scenario B and benthic in scenario C. Double lines (blue and brown) indicate a biphasic, pelago-benthic life cycle (with pelagic larval and benthic adult forms). Note that the biphasic life cycle is assumed to have evolved multiple times independently in scenario C. **(A)** The classical view implies homology of both ciliated larvae and benthic adults that, once evolved, have remained part of the eumetazoan life cycle [17]. **(B)** Nielsen [16] modified this view to propose that the holopelagic neuralian ancestors persisted beyond the initial divergence of the major neuralian clades, and that the biphasic life cycle with benthic adults arose independently in the cnidarians and once or twice in the bilaterians. **(C)** In stark contrast, other authors assume that today’s ciliated larvae arose convergently many times by the repeated intercalation of a pelagic dispersal larva into primarily monophasic, holobenthic life cycles and are thus evolutionarily unrelated. This view implies that the characteristics of today’s swimming larvae such as apical organs and equatorial ciliary bands evolved convergently [18].

The vast majority of animal phyla live in the ocean and develop via small ciliated larvae that form part of the zooplankton [1]. These larvae, called ‘primary larvae’ , are equipped with sensory cells to perceive various stimuli including light, touch and chemical cues [2,3]. Simple nervous systems integrate sensory information and control ciliary locomotion [4]. Most conspicuously, an ‘apical organ’ is found in various groups, including cnidarians [5], protostome annelids, mollusks [2], flatworms [6] and nemertines [7], as well as deuterostome echinoderms [8] and hemichordates [9]. These groups belong to the Neuralia (which includes cnidarians and bilaterian protostomes and deuterostomes; Figure 1) [10]. Apical organs are often equipped with an ‘apical tuft’ of long cilia and include a small set of sensory-neurosecretory cell types [11,12]. As shown for cnidarians, mollusks and annelids, apical organs play a role in the control of settlement [13-15], facilitating the integration of multimodal sensory input and the coordination of effector cells. Development involving primary larvae (or any other kind of secondary larvae) is referred to as ‘indirect’; if it does not involve a larval stage, it is referred to as ‘direct’ [16].

Widespread occurrence of ciliated larvae, with similar overall body plans, has prompted radically different views regarding their evolutionary significance [16,18-20]. While some authors consider them an ancient feature of the metazoan life cycle [16,17,19], others assume that they evolved multiple times independently [18,20] (Figure 1). In the past two decades, studies of developmental genes involved in setting up the larval body plan have started to provide a new source of comparative data to resolve these conflicting views. These studies have revealed specific similarities such as the expression of the *goosecoid* and *brachyury* genes in foregut, midline and hindgut of lophotrochozoan and deuterostome larvae [21] (in a manner distinct from direct developers such as insects or vertebrates). Also, the ciliary bands characteristic for swimming larvae were found to specifically express *otx* and three conserved microRNAs in lophotrochozoans and deuterostomes, corroborating the homology of shared larval features [22,23]. Conversely, dissimilar expression reported for *nk2.1* and *hnf6* in the apical plate of sea urchins and the episphere of abalone larvae [24] raised doubt regarding the common origin of similar shared larval features. However, these pioneering studies thus far relied on small gene sets.

More recently, an extended set of transcription factors has been shown to play a conserved role in patterning the larval body plan in hemichordate [25] and in sea urchin [26,27], belonging to the deuterostomes, and in the sea anemone *Nematostella vectensis*, a cnidarian [28]. These factors respond to the differential stabilization of β-catenin along the primary body axis, triggered by Wnt signaling [28,29]. In particular, *six3* and *foxq2* have been shown to negatively respond to Wnt signaling in a complex sequence of patterning events [28,29]. These factors specify apical territory around the apical pole [27,30]. For the first time, the conserved regional expression of similar sets of transcription factors provides a molecular framework for the comparison of larval cell types and tissues and thus important clues to larval body plan and apical organ evolution.

In our current study, we investigated the apical patterning system in the marine annelid *Platynereis dumerilii*[31], a lophotrochozoan protostome with a canonical biphasic pelago-benthic life cycle (involving a pelagic phase, that is, swimming, primary larvae, and a benthic phase, that is, bottom-dwelling adults). We examined the set of transcription factors involved in apical patterning, including *six3* and *foxq2*, in the episphere of the *Platynereis* trochophore larva and have shown that, as in deuterostomes and cnidarians, expression of these factors is sensitive to Wnt signaling. We found that the apical organ develops in a small central territory devoid of *six3* expression, that instead expresses a number of other factors, many of which are found in the same location in other neuralians. By expression profiling, we molecularly characterized several cell types that form part of the apical organ in *Platynereis*, which we compared to apical organ cell types described for other animal groups.

Our results reveal that the larvae of cnidarians, protostomes and deuterostomes exhibit extensive similarity in the molecular topography of body regions around the apical organ, which we use to genetically define ‘apical plate’ and ‘apical organ’; the specification of these regions by a conserved apical signaling system; and the molecular fingerprint of a subset of apical organ cell types. These findings support homology of some primitive type of apical organ (and thus of swimming ciliated larvae) in Neuralia and are most consistent with an early and unique origin of animal larval forms (Figure 1A,B).

## Results

### Molecular topography of the apical region

In the sea urchin [26,27], the hemichordate *Saccoglossus kowalevskii*[25] and the anthozoan cnidarian *Nematostella*[28,30], expression of the transcription factors *six3* and *foxq2* demarcates the most apical body region. In *Saccoglossus* and *Nematostella*, the *six3+*, *foxq2+* domain is peripherally overlapping with a ring of *rx* and bounded by even more peripheral rings of *otx* and *otp* expression [25,28]. Likewise, *fezf* is expressed in the apical plate in sea urchin [32] and the hemichordate [33]. We have previously shown that *six3* is expressed in a large contiguous domain of the *Platynereis* episphere [34], peripherally overlapping with the expression of *rx*[35].

**Figure 2:**
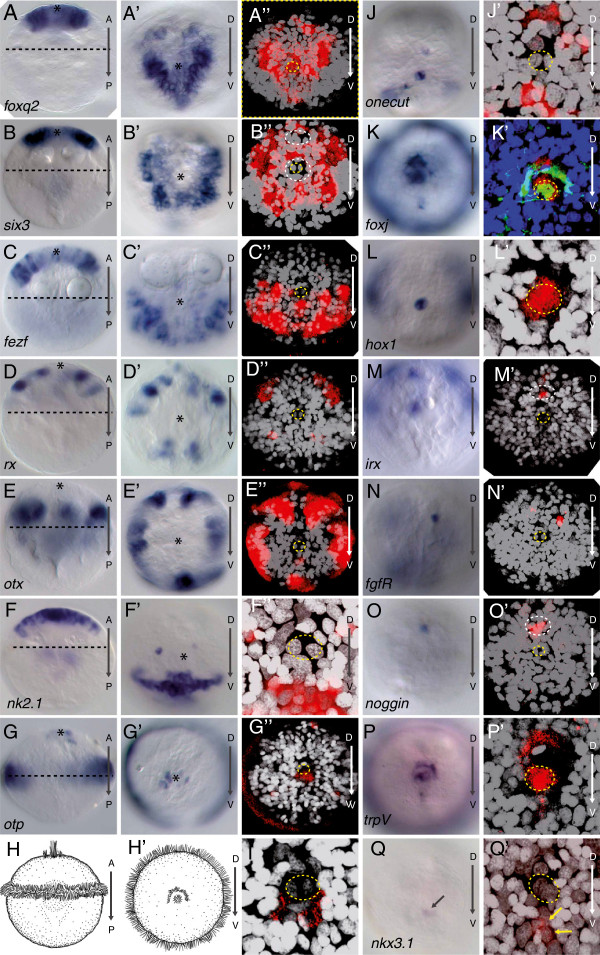
**Apical regional patterning mRNA expression in early trochophore larvae. (A-G)** are ventral views, **(A’-G’)** and **(J-Q)** are apical views, and **(A”-G”)** and **(J’-Q’)** are apical views using confocal reflection microscopy of NBT/BCIP staining (red) following *in situ* mRNA and fluorescent DAPI staining of nuclei (white), allowing localization of individual cells in the apical Plate. F”, I, J’, K’, L’, P’ and Q’ are high magnification apical views. Ampullary cells are demarcated with yellow dashed circle. All images are of 24 hour post-fertilization (hpf) embryos, except C, C’, D, D’, E, E’, G, G’, L, M, O, O’, which are of 20 hpf embryos. Asterisks mark the tip of the apical pole. **(A)** Broad apical expression of *foxq2* in 24 hpf trochophores. **(B)** Broad apical expression of *six3*, excluding area encompassing apical organ and a dorsal patch of cells (demarcated with white dashed lines). **(C) ***Fezf* ventrally in apical plate. **(D) ***Rx* in dorsal and ventral domains between the *six3* domain and the prototroch. **(E) ***Otx* in ring-like domain adjacent to prototroch. **(F) ***Nk2.1* in a ventral strip of cells and in two spots flanking the crescent cells. **(G, I) ***Otp* in apical cluster and in prototroch. **(H, H’)** Schematized ventral and apical views at 24 hpf depicting crescent and ampullary cells and prototroch. **(J) ***Onecut* in crescent cells and in cells ventral to the ampullary cells. **(K) ***FoxJ* in the ampullary cells, crescent cells and prototroch. **(K**’**)** Antibody staining to acetylated tubulin (green) and DAPI-labeled nuclei (blue). **(L) ***Hox1* in ampullary cells. **(M) ***Irx* in *six3*-negative territory and in an equatorial ring below the prototroch. **(N) ***FgfR* in a cell dorsal to the ampullary cells. **(O) ***Noggin* in a cell dorsal to the ampullary cells. **(P) ***trpV* in cells ventral to the ampullary cells. **(Q) ***Nkx3* in a small patch of cells ventral to the ampullary cells. (**Q’**) *Nkx3* in mechanoreceptor cells (yellow arrows).

Building on this, we set out to further refine the molecular topography of the *Platynereis* larval episphere. We found that, in *Platynereis*, the newly characterized *foxq2* expression demarcated the ‘upper’ two thirds of the episphere (Figure 2A), largely overlapping with that of *six3* (Figure 2B). We refer to this *six3+, foxq2+* region as apical plate, in accordance with classical nomenclature for spiralian trochophore larvae (for example, see [2] and discussion). Expression of the newly characterized *fezf* (Figure 2C) and that of *rx* (Figure 2D) overlapped that of *six3* and *foxq2* peripherally (with *rx* being largely restricted to the dorsal and *fezf* to the ventral body sides). More peripherally, in the ‘lower’ third of the episphere, we detected expression of *otx* (Figure 2E) [21], partially overlapping with that of *rx* dorsally and that of *fezf* ventrally. Even more peripherally, expression of the *otp* gene (Figure 2G) [12] demarcated the ciliary band, called the prototroch.

**Figure 3:**
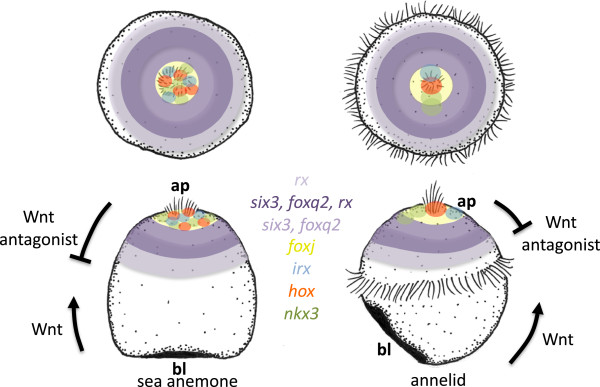
**Comparison of apical molecular territories in a sea anemone planula larva and an annelid trochophore.** Apical (above) and lateral (below) views of a schematized planula larva of a sea anemone (left panel). Gene expression based on published data for *Nematostella vectensis* (for references see text). Apical (above) and lateral (below) views of a schematized annelid trochophore larva (right panel). Gene expression based on our data.

The *Platynereis* episphere can thus be subdivided into a sequence of molecular regions, arranged in concentric rings from apical to peripheral (Figure 3). This overall sequence matches the molecular topography observed in deuterostomes and cnidarians (Figure 3)*.* Another conserved feature of apical patterning in sea urchin [24,36], *Saccoglossus*[25] and again in *Platynereis*[12] is expression of *nk2.1* in an apical-ventral territory (Figure 2F), overlapping *six3*. In sea urchin, this domain includes cells positive for *onecut* (*HNF6*) [24]*,* and we found that, in *Platynereis*, expression of the same genes covers cells located ventromedially at a similar distance to the apical tuft cells (demarcated by a yellow dotted line in the confocal reflection microscopy panels; compare Figure 2F” with Figure 2J’), which indicates co-expression. This is important because the two genes were shown to comprise non-overlapping domains in the apical region of the red abalone *Haliotis* (where *onecut* is expressed dorsal to the apical organ only [24]; note that *Platynereis* also shows dorsal patches of *onecut* expression in Figure 2J’).

The *Platynereis* trochophore also shares expression features with cnidarian and deuterostome larvae specific to the developing apical organ. In sea urchin [37], *Nematostella* and *Platynereis*, the apical tuft and a girdle of cells surrounding the tuft are devoid of *six3* expression (white dashed line in Figure 2B’ and compare to Figure 3 in Poustka *et al*. [37] and Figure 3d in Marlow *et al*. [28]). In *Platynereis*, the *six3-*free region extends into a dorsal patch of cells that becomes separated from the central patch by a band of *six3*+ cells (which includes the conspicuous crescent cells, see below). Inside the *six3-*free region, we detect restricted expression of several transcription factors (using the nuclei of the two apical tuft cells for reference; yellow circles in confocal apical views in Figure 2): *otp* (Figure 2G”; in addition to its ring-like expression, see above), *nkx3* (Figure 2Q’), *foxj* (Figure 2K’) and *hox1* (Figure 2L’). In addition, the *irx* gene is expressed in the dorsal patch of cells devoid of *six3* expression (Figure 2M’). Notably, *nkx3*[28]*, foxj*[30], the *hox* gene paralog *anthox1*[28] and *irx*[28] are also expressed in the apical region devoid of *six3* in *Nematostella*; *foxj* matches the region devoid of *six3* expression in sea urchin [38] and apical *irx* expression has been documented in hemichordates, although broader than in annelids [25]. An apical region devoid of *six3* expression, ‘filled’ by the restricted expression of *foxj, nkx3*, *hox* and *irx*, thus represents a larval-specific transcription factor signature that molecularly defines the apical organ in several distinct, phylogenetically remote neuralian groups such as anemone and annelid (Figure 3) and lends strong support to its evolutionary conservation (see Discussion).

**Figure 4:**
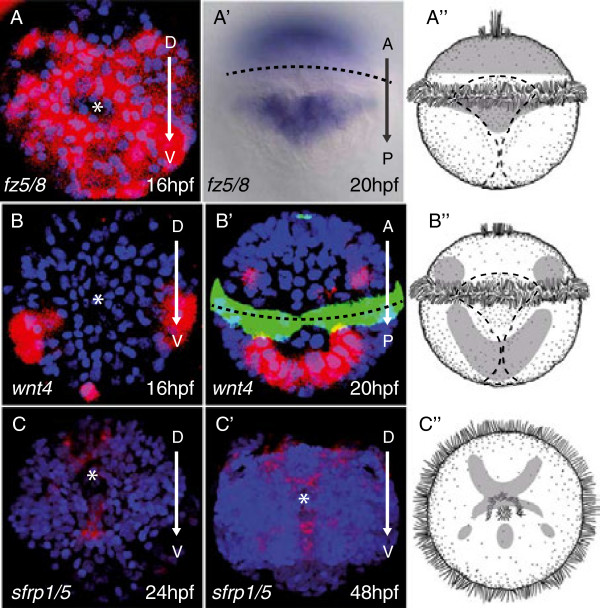
**Wnt pathway members are expressed in the episphere and hyposphere during early development.** The prototroch is marked with a dashed line. The tip of the apical plate is marked with an asterisk. Arrows indicate dorsal (D), ventral (V), anterior (A) and posterior (P). **(A) ***Frizzled5/8* across the apical plate, 16 hours post-fertilization (hpf) apical view and a **(A’)** 20 hpf ventral view (inset shows a surface ventral view). **(B) ***Wnt4* along the blastopore, 16 hpf lateral view. **(B’)** Confocal image of ventral view of Wnt4 expression in which antibody staining to acetylated tubulin is visible with a green secondary antibody, reflection signal of *in situ* hybridization is red and nuclei are visible, stained with DAPI, in blue. **(C)** Apical view of *sfrp1/5* expression at 24 hpf and at **(C’)** 48 hpf. **(A”, B” and C”)** Schematic representations of the expression patterns in **(A)** and **(B)** and **(C)** and **(A’)** and **(B’)** and **(C’)**.

Functional studies have demonstrated the role of fibroblast growth factor (Fgf), transforming growth factor (Tgfβ and Fgf signaling) and Wnt signaling in apical plate patterning. For example, Fgf signaling controls formation of the apical organ in sea anemone [14]; the apical plate of sea urchins is patterned by Tgfβ signaling [39] and *fgfr1* localizes to the apical plate during apical organ formation [37]. Similarly, the *Platynereis* apical organ region showed specific expression of *fgfr* in a highly restricted population of cells (Figure 2N). We also found an apically restricted domain of the Tgfβ signaling antagonist *noggin* in cells devoid of *six3* expression (white dashed line in Figure 2O’), which indicates that both signaling systems also play a role in apical patterning in *Platynereis.* Notably, expression of *noggin* has also been reported for the *six3*-free apical organ in sea anemone [40]. Consistent with a prominent role of Wnt signaling in *Platynereis* apical patterning, we detected the Wnt receptor molecule *frizzled5/8* in a broad apical domain (Figure 4A; as seen in the hemichordate *Ptychodera*[41] and sea urchin [42]) and *sfrp*, which plays a conserved role in antagonizing Wnt signaling in deuterostomes [42,43], specifically in the apical organ region (Figure 4C,C’ ,C”). Furthermore, we detected early expression of *wnt4* in ventral peripheral episphere regions, outside of (but abutting) the *six3* domain, consistent with a role in apical patterning (Figure 4B,B’). *Wnt4* is one example of a Wnt paralog expressed at the right stage and location in developing *Nematostella*[44] and amphioxus [45] to be involved in patterning along the primary axis; yet, in the absence of specific knockdown or knockout data, no case for the specific involvement of Wnt4 can be made. Note that none of the many Wnts expressed in *Platynereis* larvae [46,47] appears to be expressed in the *six3+* apical plate, in line with repression of Wnt signaling being a pre-requisite for apical specification to occur, a notion that we set out to test further experimentally.

### Ectopic Wnt signaling abolishes specification of the apical organ region

**Figure 5:**
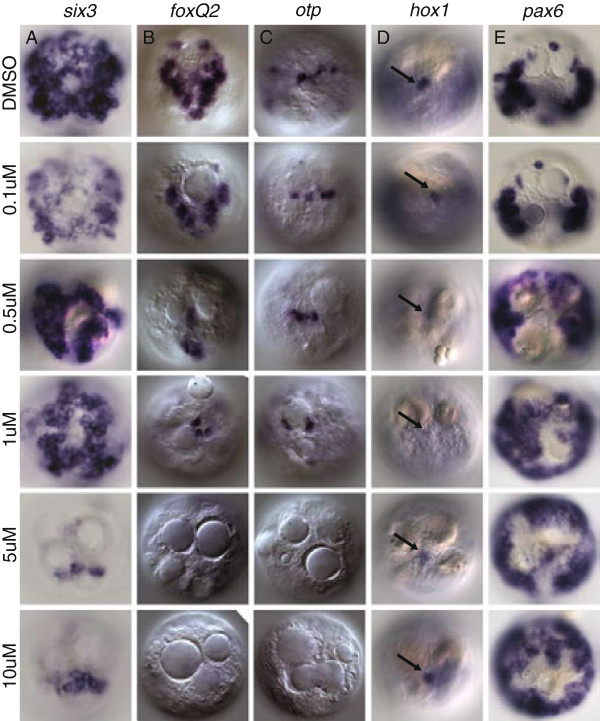
**Azakenpaullone treatment of embryos alters gene expression in the episphere in a dose-dependent manner.** All images are apical views with dorsal at the top of the image and ventral at the bottom. **(A) ***Six3* is severely reduced in embryos treated with azakenpaullone. **(B) ***Foxq2* expression is lost in all but the most apical cells of the episphere in azakenpaullone treatments. **(C)** Fewer *otp* cells are stained at increasing concentrations of azakenpaullone and expression is entirely lost at 10 μM. **(D) ***Hox1*, a molecular marker of the ampullary cells (arrows), is not affected by azakenpaullone treatment. Ampullary cells are specified very early in development, earlier than the initiation of chemical treatments at 12 hours post-fertilization, prior to the differentiation of the remainder of the episphere. **(E) ***Pax6* expression, which marks the ventral regions of the developing apical plate, is expanded in the episphere upon treatment with azakenpaullone. Counts of the embryos displaying wild-type, reduced or expanded expression domains for two replicates at 0, 1 and 5 μM concentrations are displayed in Figure S4.

**Figure 6:**
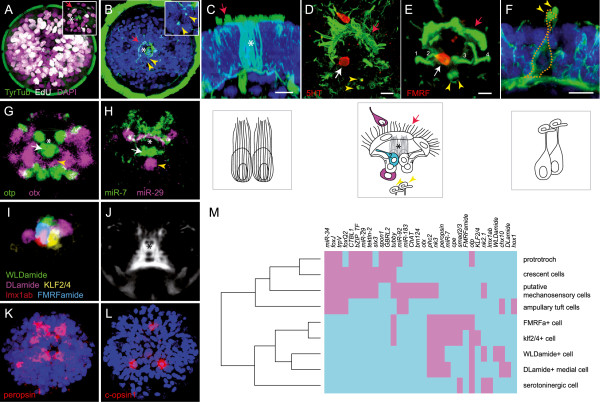
**Molecular fingerprint of *Platynereis *apical organ cell types. (A)** EdU incorporation (white) in dividing cells between 22 and 24 hours post-fertilization (hpf) (apical view). Central EdU-negative cells (magenta) represent post-mitotic apical organ cells. Inset: 5 μm z-projection of apical organ. **(B)** Acetylated tubulin staining at 30 hpf, apical view. Crescent cells located dorsal to ampullary tuft cells. Inset: Cilia of the putative mechanosensory cells (arrowhead). **(C)** Lateral view of ampullary tuft cells, acetylated tubulin (green) and DAPI staining (blue), 30 hpf. **(D)** Serotoninergic cells of the apical organ stained with an anti-5HT antibody, 48 hpf. White arrow indicates serotonergic interneuron (magenta in schematic). **(E)** Four *otp* + peptidergic flask-shaped cell (numbers) at 48 hpf (apical view). One of these labeled by an FMRFamide antibody. In schematic, numbers indicate *otp +* cells and the FMRFamidergic cell is in cyan. **(F)** Lateral view of putative mechanosensory cells, 48 hpf (arrowheads: stiff curly tips of the sensory cilia). **(G, H)** Mutually exclusive gene expression at 48 hpf, 23 μm z-projection of averaged expression patterns obtained with profiling by image registration (PrImR). **(I)** Cell types of the *otp* + domain. The image shows non-overlapping expression of markers of each cell as deduced from PrImR, 11 μm z-projection of average expression patterns. **(J)** 4 μm z-projection of the average axonal scaffold at 48 hpf, showing the deep position of the two ampullary cells surrounded by neuropil. **(K)** Expression of *peropsin* at 24 hpf **(L)** Expression of *c-opsin1* at 20 hpf. **(M)** Hierarchical clustering of the molecular fingerprint of individual cell types identified within the apical organ region and of the prototroch for comparison. Expression in magenta. Asterisk indicates the two ampullary tuft cells, red arrows indicate crescent cells and yellow arrowheads indicate cilia of two putative mechanosensory cells. In panels B-H, gene expression is in red, DAPI staining in blue and tubulin staining in green unless otherwise specified. Panels C-F, scale bar is 10 μm.

In deuterostome larvae such as sea urchin and *Saccoglossus*, as well as in the cnidarian *Nematostella,* the expression of apical markers such as *foxq2* and *six3* critically depends on levels of active Wnt signaling [27,43,48]. Activation of canonical Wnt signaling via chemical inhibition of Gsk3β via Li + and/or paullone treatment in sea urchins [37], hemichordates [43] and cnidarians [28] results in the loss of apical ectodermal markers. Conversely, degradation of nuclear β-catenin in sea urchin leads to expansion of apical ectodermal genes including *nk2.1*, *foxq2*, *six3*, *rx* and *fgfR*[27], indicating that Wnt-mediated antagonism of apical plate markers plays a role in the development of apical territories in deuterostome and cnidarian larvae. To test the role of Wnt signaling in apical patterning in *Platynereis* larvae, we exposed early trochophores to azakenpaullone, a selective inhibitor of Gsk3β [49] that has been shown to trigger nuclear β-catenin accumulation in *Platynereis*, mimicking ectopic activation of Wnt signaling [47,50]. We found that azakenpaullone specifically knocked down or abolished apical expression of episphere markers, including the broadly expressed *six3* (Figure 5A) and *foxq2* (Figure 5B) in a concentration-dependent manner, with the majority of expression reduced or lost between 1 μM and 5 μM (Figure 5). Conversely, the expression of *pax6*, which occupies a more ventral peripheral position in the larval episphere [51] (overlapping with Wnt4; Figure 4E), was expanded at 0.5 μM to 10 μM concentrations of azakenpaullone (Figure 5E). The number of *otp +* apical organ cells was also reduced at higher concentrations of azakenpaullone (Figure 5C), with all cells absent at 5 and 10 μM concentrations. By contrast, the expression of *hox1* in the apical tuft cells persisted at all concentrations (Figure 5D, arrows). This may have been because tuft cells are among the first cells to differentiate in the apical plate (Figure 6A). To test the dynamic role of Wnt signaling in episphere patterning, we conducted washout experiments in azakenpaullone-treated embryos (Additional file 1: Figure S3). Following washout at 24 hours, embryos were assessed at 30 hours post-fertilization (hpf) for recovery of gene expression. We saw moderate recovery in the expression of *foxq2* and *six3* as well as a slight restriction of *pax6*, but no change in the number and location of *otp +* cells. We attribute the moderate recovery in expression to the determinate lineage of *Platynereis* larvae, in which stereotyped divisions may result in a restricted fate potential very early in development. Taken together, our data indicate that in *Platynereis* as in deuterostome larvae, the transcription factors defining the molecular identity of apical body regions, of which *six3* and *foxq2* form core components, are opposed by a Wnt-dependent signaling center.

### Morphological and molecular characterization of apical organ cell types

Light and electron optic studies have revealed cell types that make up the apical organs of different invertebrate larval groups [11,52-54]. In *Platynereis*, the apical tuft was visible by 16 hpf (cf. *trpV* channel expression at 24 hpf, Figure 2P) and other apical organ cells were likewise post-mitotic by 24 hpf (Figure 6A), expressing markers indicative of neuronal differentiation. At 30 hpf, several apical cell types were fully morphologically differentiated (Figure 6B; [31]). We found that the apical tuft was formed by two basket-shaped cells with intracellular tubulin support structures (Figure 6C); cells with a very similar morphology, referred to as ampullary cells, have previously been described in mollusk larvae [11]. These cells persisted deep in the medial brain at later stages in the center of a massive commissural and neurosecretory neuropil (Figure 6J), and may thus represent a structural organizing center for the juvenile nervous system, as suggested for other polychaete larvae [50]. Dorsal to the ampullary tuft cells, we found another set of large cells with multiple motile cilia in a crescent-moon shape, known as crescent cells (Figure 6D). Two serotonergic cells have also been found in the apical organ region by 30 hpf [31]. Closest to the tuft was a serotonergic interneuron (white arrow, Figure 6D) lacking sensory dendrites. This cell was located deep in the epithelium, adjacent to an assembly of previously described sensory-neurosecretory flask-shaped cells (Figure 6E) that, morphologically, resemble chemosensory cells [12] (called parampullary cells in mollusks [11]). More ventral to the parampullary cells, we detected a median pair of cells bearing short, stiff and curly sensory cilia resembling mechanoreceptors (Figure 6F).

These distinctive morphologies, in conjunction with the recently established Profiling by Image Registration (PrImR) technique, enabled us to assign a molecular fingerprint to these cells, providing them with unique molecular identities [47]. PrImR utilizes the stereotyped development of the *Platynereis* axonal scaffold to generate *in silico* alignments of mRNA *in situ* expression patterns, and allows single cell co-expression analyses to be conducted [47]. Of a collection of 140 genes currently available for PrImR single cell co-expression analysis, 29 were differentially expressed in cells of the apical organ region in the 48 hpf larva. Additional file 1: Figure S5 details the PrImR-based co-expression analysis for the above-mentioned morphologically identifiable cells, namely the ventral-most serotoninergic cell (Additional file 1: Figure S5A), the parampullary sensory-neurosecretory cells (Additional file 1: Figure S5B-E), the ampullary tuft cell (Additional file 1: Figure S4F), the crescent cells (Additional file 1: Figure S5G) and the pair of putative mechanoreceptors (Additional file 1: Figure S5H).

PrImR revealed unique sets of genes expressed by each of these cell types, in line with their specialized sensory-neurosecretory and neuronal characteristics. For example, as determined previously, the flask-shaped parampullary cells expresses *otp* (Figure 6G; Additional file 1: Figure S5B-E; and see above), *mir-7* (Figure 6H) and *prohormone convertase 2* (*phc2*) [12]. Beyond that, PrImR allowed cellular allocation of transcripts encoding neuropeptide precursors for DLamide, FMRFamide and WLDamide (Figure 6I), consistent with a conserved role of otp in specifying different types of peptidergic cells [12]. These three neuropeptides mediate opposing effects on locomotor behavior with DLamide and FMRFamide exposure leading to an increase and WLDamide leading to a decrease in ciliary beating frequency [55]. The *otp +* peptidergic cells also expressed MIP, the recently described settlement-inducing neuropeptide [15]. In addition, we found that all *otp +* sensory-neurosecretory cells were positive for the newly identified *peropsin* gene (Figure 6K), an opsin-related photopigment that may function as photopigment or photoisomerase [56], indicating that these cells (or adjacent cells) are light-sensitive. Complementing this, we observed that the previously characterized *c-opsin1*[35], an ortholog of *rhodopsin* and other *c-opsins*, was expressed in cells around the apical organ (Figure 6L). As *c-opsin1* expression is difficult to score in 48 hpf larvae (the stage for which the PrImR resource is available), we were not able to further characterize the *c-opsin1+* cells. One of the *otp +* cells was demarcated by the expression of *lmx1ab* (a terminal selector gene for serotoninergic neurons [57]) and by position correlates to the serotonergic interneuron (Additional file 1: Figure S5A; Figure 6I). The ampullary apical tuft cells (Additional file 1: Figure S4F) specifically expressed *hox1* (Figure 2L) and a trpV channel previously implicated to serve mechanosensory roles in other protostomes (Figure 2P) [58], indicative of multiple sensory modalities. Finally, and consistent with their specialized morphology, the two putative mechanosensory cells (Additional file 1: Figure S5H) expressed *miR-183* (Additional file 1: Figure S1), a conserved microRNA that demarcates chemo- and mechanosensory cells across bilaterians [22,59,60]. Furthermore, they were the only cells to express *otx* (compare Figure 2E’).

### Hierarchical clustering reveals distinct groups of apical organ cells

In line with our observation that at 24 hpf the apical organ region including the tuft cells was devoid of *six3* expression, the tuft cells themselves and the directly ventrally adjacent sensory-neurosecretory and serotoninergic cells were *six3-*negative at 48 hpf. By contrast, the crescent and mechanoreceptor cells expressed *six3* and thus, by molecular identity, appear to represent differentiated cell types of the surrounding apical plate. Hierarchical clustering based on the 48 hpf PrImR data (Figure 6M) supports this distinction: one cluster comprised the flask-shaped sensory-neurosecretory cells and the adjacent serotoninergic cell, which, in addition to the absence of *six3* and presence of *otp*, *miR-7* and *phc2* (see above), expressed *carboxypeptidase-E* (*cpe*) and other genes not directly linked to neurosecretion, such as *smad2/3*. This cluster corresponded to the *otp +* cells devoid of *six3* expression at 24 hpf (see above). Another well-supported cluster comprised the crescent cells and the putative mechanosensory cells, which, in addition to *six3*, expressed the microRNA miR-29 (Figure 6H), the *ctbl1* and *bZIP.TF* genes encoding transcription factors of unknown function, and *tektin-2*, a structural component of microtubules [61]. Note that in our clustering, absence and presence of *six3* and *foxq2* strictly correlated, indicative of co-regulation [30] (Figure 6M).

The ampullary tuft cells devoid of *six3* (and *foxq2*) exhibited a more separate identity with equal distance to either cluster. With the *six +* (and *foxq2+*) mechanosensory and crescent cells, they shared expression of *foxJ*, which plays a role in the formation of long motile cilia [62], and of three microRNAs of the ‘ciliary’ group that have previously been found to demarcate locomotory ciliary bands across Bilateria [22]. They shared the expression of the transcription *chx10* with the peptidergic cells (Figure 6M).

## Discussion

### Evolutionary conservation of apical patterning

**Figure 7:**
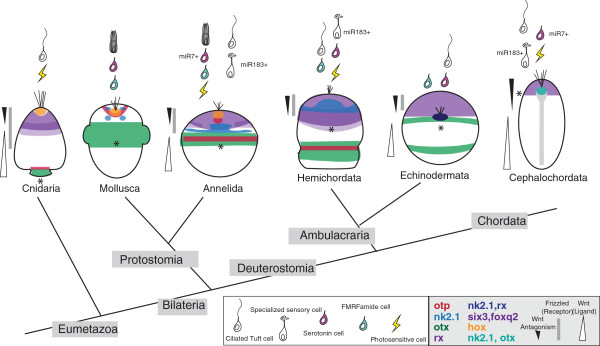
**Larval molecular territories and cell types show global conservation of ancient larval patterning.** For animals in which molecular data is available, spatial transcription factor expression data have been mapped onto the larval body plan (larvae as seen from the ventral side; amphioxus is pictured from a dorsal perspective). Morphologically described or ‘molecularly footprinted’ cells found in the apical organ are pictured above larval forms. The activity of Wnt signaling on larval body formation is pictured with bars flanking the larvae. Data is compiled from this and previous studies in Cephalochordata [45,59,65-73], Hemichordata [9,25,41,43,60,74-77], Echinodermata [26,32,36,37,42,48,78-83], Mollusca [24,84] and Cnidaria [30,40,44,64,85-90].

We detected strong similarities in molecular topography around the apical organ of ciliated larvae in protostomes, deuterostomes and cnidarians that we interpret as common heritage. It is highly unlikely that a specific and partially nested pattern involving at least nine transcription factors (*rx*, *six3*, *foxq2*, *foxj*, *otx*, *otp*, *irx*, *nkx3, hox*) evolves convergently twice. The core of this pattern that is shared between all neuralians appears to be the apical co-expression of two factors, *six3* and *foxq2*, in sea urchin [26,27], hemichordate [25], annelid (our data), brachiopod [63], sea anemone [28,30] and hydrozoan [64]. In sea urchin and spiralian larvae, these factors demarcate the apical plate and we propose to expand this term to the *six3+*, *foxq2+* region of all neuralian primary larvae, which should be universally referred to as the apical plate (purple in Figure 7). More peripherally, the apical plate co-expresses *rx*, as demonstrated here for the annelid but as also found in the sea anemone [28] and hemichordate [25] (dark purple in Figure 7). (In sea urchin, *rx* appears to be expressed in the entire apical plate.) In all neuralians investigated, the specification of the apical plate is sensitive to Wnt signaling, in that pharmacological activation of Wnt signaling abolishes the expression of *six3*, *foxq2* and of other apical markers ([27,28,43,48] and our data). The gene regulatory network establishing *six3* and *foxq2* expression is only beginning to be elucidated and appears to commonly involve activation of *foxq2* by *six3*[30]. In bilaterians, the apical plate also appears to peripherally co-express *fezf* (in annelid, sea urchin and in *Saccoglossus*) and ventrally *nk2.1* and *hnf6/onecut* (in annelid and in sea urchin; blue in Figure 7).

Notably, although the *six3+*, *foxq2+*, *rx+*, *fezf+*, *nk2.1*, *onecut +* apical plate, as defined here, is a larval character, the same genes are also co-expressed in bilaterian phyla that have lost the primary larva, such as insects and vertebrates, where they specify anterior brain regions [34,91,92]. This is consistent with previous observations that, after *Platynereis* metamorphosis, apical plate markers remain expressed and demarcate the developing cerebral ganglia of the adult nervous system [12]. Therefore, while the stereotypical, partially overlapping co-expression of the above-mentioned genes can be used to topographically ‘align’ larval body plans, it is not a unique feature of primary ciliated larvae.

### A universal molecular definition of the apical organ region

The apical organ develops in the center of the apical plate. In annelid, sea urchin [37] and sea anemone [28,30], this apical organ region is specifically excluded from the region of *six3* expression (in annelid (Figure 3A) and cnidarian (Figure 3C) [30]). Instead, it expresses the transcription factors *foxj* (in sea anemone [30] and sea urchin [38]), *nkx3* (in annelid (Figure 2Q) and sea anemone [28]), *irx* (in annelid (Figure 2M) and sea anemone [28]) and a *hox* paralog (in annelid (Figure 2L), mollusk [93] and sea anemone [28]). Note that all of these factors are also expressed elsewhere; for example, in *Platynereis*, *foxj* is expressed in other *six3+* ciliated apical plate cells and, more generally, in ciliary bands; *nkx3* is expressed in other apical plate cells (our data) and in segmented mesoderm [94] and *hox1* is expressed in the second larval segment [95] - nevertheless, the recurrent appearance of these factors in the apical organ region across neuralians appears highly significant and we propose that it reflects the evolutionary conservation of apical organ cell types (see below). In addition, apical organ formation appears to similarly depend on local FGF signaling, as shown for sea anemone [14] and as suggested by localization of the *fgf* receptor to the apical plate, in sea urchin [37] and annelid (Figure 2N). Also, the common localization of Tgfβ signaling inhibitor *noggin* to the apical organ, as seen in sea anemone [40], sea urchin and in *Platynereis*, is indicative of conserved signaling events. Together, these transcription factors and signaling molecules provide a highly characteristic molecular signature for the apical organ region (and the cell types therein, see below). Given that the establishment of the *six3*-‘hole’ spatially correlates with, and has been functionally linked to, the formation of the apical tuft (see below), we consider this signature a characteristic feature of primary larvae and its apparent evolutionary conservation lends strong support to the notion that these larvae represent an ancient feature of the metazoan life cycle (Figure 1A and see above). It will be interesting to determine to what extent this signature or parts of this signature are present in groups that have lost primary larvae; given the spotted appearance of *irx*, *nkx3*, *hox* and *foxj* expression in the apical organ region it is likely that any conservation of this signature at adult stages would relate to the persistence of ‘apical’ cell types throughout the life cycle.

It should be stressed that the *hox* genes expressed in the apical organ in cnidarian, mollusk and annelid represent different paralogs of the Hox cluster. *Anthox1*, which demarcates the apical organ in sea anemone, is a ‘posterior’ *hox* gene [96]; *Lox5*, *Lox4* and *Lox2*, which are expressed in different cells of the apical organ of the snail trochophore [93], belong to the middle part of the cluster; and *hox1*, that we find expressed in the *Platynereis* apical organ, is an ‘anterior’ *hox* gene. The utilization of different *hox* paralogs in the apical organ can be explained if one assumes that *hox* expression in apical organ cells is older than the *hox* cluster itself and already occurred at the times when a single ur-*hox/parahox* gene existed [97]. Then, concomitant with subsequent duplication events, the expression of different *hox* paralogs would have been lost in divergent evolutionary lines. Alternately, the *hox* paralogs utilized in the apical organ may have been exchanged over time. Additional studies examining the downstream targets of hox expression will be useful in distinguishing between these scenarios.

### The apical tuft - ancient nucleation center of apical organs

If the neuralian ancestors specifically expressed the molecular signature defined above in selected cells of the apical organ region, what cell types did the partaking genes specify and what can we learn about the ancestral composition and function of the neuralian apical organ? Except for *Platynereis*, cellular resolution molecular fingerprints are not yet available in marine primary larvae, limiting the extent of interphyletic comparison of specific cell types. However, limited molecular evidence is available and adds to a rich body of morphological evidence. The first and most obvious candidates for conserved apical organ cell types are the apical tuft cells that are widespread in Neuralia [2,11,85,98]. In *Platynereis*, the apical tuft cell exhibits a basket-shaped morphology with characteristic intracellular support structures resembling those of ampullary tuft cells in mollusks [11] and stands out with the specific expression of *hox1*. In the snail *Gibbula*, expression of *Lox4* is confined to ‘two cilia-bearing cells of the apical organ’ [93]; it is as yet unclear how these relate to the ampullary tuft cells. In sea anemone, injection of *anthox1* morpholino results in planulae lacking the apical tuft [30], corroborating a link between *hox* expression and tuft formation. Given that *anthox1* knockdown also abolishes the ‘hole’ in *six3* expression, one might speculate that the formation of an apical tuft involving restricted *hox* expression, FGF signaling and apical down-regulation of *six3* represents the conserved core of apical organs in Neuralia (see above). This notion would imply, however, a transition from monociliated to multiciliated tuft cells: while the *Platynereis* apical tuft cell bears multiple long motile cilia, those of some other spiralian and of deuterostome larvae are monociliated [74]; likewise, the cnidarian apical tuft is composed of many cilia that each emerge from single monociliated cells [5]. These long cilia perform a mechanosensory function during sweeping behavior of the benthos [99,100]. In the absence of cell type-specific expression data from other neruralian larvae, the evolutionary relationship of the various tuft cells remains speculative; future studies will further clarify their evolutionary relationships within neuralians.

### The apical plexus - a conserved sensory-neurosecretory release site for the control of body physiology and metamorphosis

Besides the apical tuft, the apical plexus directly underneath the apical tuft may belong to the ground pattern of apical organ structures in Neuralia, as it is commonly innervated by flask-shaped, neuropeptidergic sensory-neurosecretory cells from the apical plate or apical organ proper - in mollusks [101,102], sea urchins [78], hemichordates [74] and, likewise, cnidarians [85,103-107]. Among the neuropeptides secreted by these cells into the apical plexus may be two ancient types of short amidated peptides, Wamides and R(F/Y)amides, recently proposed to predate neuralians [108]. In cnidarians, RFamidergic sensory neurons from ‘anterior’ larval regions project into the apical plexus [85,105,106,109]. Likewise, RFamidergic cells populate the apical plate and organ in annelids (as shown here and in [12,110]), mollusks [101,102], polyclad flatworms [6] and phoronids [111]. Wamides have been implicated in the control of larval settlement in cnidarians [112,113] and, recently, in annelids [15] and are likewise released from the apical plexus. In the late planula larva of the hydrozoan *Hydractinia*, GLWamide-positive sensory cells that innervate the apical plexus populate a belt around the apical pole [114], which, by position, forms part of the apical plate; MIP, a related Wamide neuropeptide that induces larval settlement in annelids, is secreted into the apical plexus by apical organ cells [15].

In *Platynereis*, the transcription factor *otp* demarcates the neuropeptidergic sensory-neurosecretory cells in apical organ (Figure 2G,G’ ,G” and [12]). Since *otp +* cells also exist in the apical organ of the snail *Patella*[84] and are overlapping with *six3* expression in the apical plate of the late *Nematostella* planula [28], we hypothesize that *otp* + sensory-neurosecretory cells projecting to the apical plexus formed part of the apical plate in neuralian ancestors and that subsets of these cells became part of the apical organ in the bilaterian lineage. Another gene demarcating the neuropeptidergic sensory-neurosecretory cells, among other cells, is *nkx3*. The apparent deep conservation of *nkx3* expression in apical organs (see above) suggests an ancient role of *nkx3* in the specification of these apical neurons; to find out, a thorough investigation of *nkx3+* cells in other apical organs, for example in the cnidarian planula, will be rewarding.

Besides the neuropeptidergic cells, apical serotoninergic cells represent a third type of neurosecretory cell projecting into the apical plexus. They exist in mollusks [11], echinoderms [78], enteropneusts [98] and other bilaterian phyla [115]. Serotonergic cells are also enriched in apical body regions of the hydrozoan cnidarian *Phialidium gregarium*, where the release of serotonin has been reported to trigger larval settlement [116]. The molecular identity of serotoninergic apical cells is beginning to be elucidated: the 5HT cell included in our PrImR analysis appears to specifically express *lmx1ab*, a LIM homeodomain factor implicated in serotoninergic specification in nematodes [57], and *nk2.1*, demarcating serotoninergic cells in sea urchin [36] and, possibly, in the *Ptychodera* tornaria [75]. Likewise, *fezf* has been proposed to correlate with serotoninergic fate in the sea urchin [32]. Further genetic studies on the serotonergic system in various marine larvae will be needed to resolve this issue.

### Ambient light detection

Finally, our study provides strong evidence for photosensitivity being an ancient feature of apical organs. Remarkably, the opsins identified in cells in and around the apical organ in *Platynereis* (Figure 6K,L), and also in *Terebratalia transversa*[117] and *Nematostella vectensis*[86], all fall within the peropsin/ciliary opsin families. These observations indicate that apical organs evolved as multimodal sensory structures, of which photosensitivity formed a key component.

### ‘Minimally indirect development’ links apical patterning of larval and adult stages

The continuous deployment of the apical patterning system at larval and adult stages and the persistence of some apical plate and organ cell types into post-metamorphic stages would suggest that a gradual type of metamorphosis (where the bulk of tissues persist, see below) is more ancient than the ‘catastrophic’ mode of metamorphosis dubbed maximal indirect development [19]. We refer to such a biphasic life cycle, with gradual and limited metamorphosis in which larval neural structures are incorporated into the adult nervous system, as ‘minimally indirect development.’ Illustrating this, the *Platynereis* apical organ tuft cell appears to form a ‘nucleation center’ around which the brain is organized (Figure 6I), and the larval axons pioneer the tracts and nerves of the later nervous system [31,54]. It is possible that the eumetazoan common ancestor showed minimally indirect development with a larval stage resembling the primary ciliary larvae of modern marine bilaterians and cnidarians.

## Conclusions

We have investigated regionalization of the larval episphere, the effects of ectopic activation of Wnt signaling on apical patterning, and the molecular fingerprint of apical cell types in the marine annelid *Platynereis dumerilii*. Comparing our findings to those in other marine larvae, we present a core set of characteristics common to primary ciliated larvae in bilaterians and cnidarians. All larvae develop an apical plate that we define by a combination of transcription factors most prominently involving *six3* and *foxq2*. Expression of these factors and formation of the apical plate is sensitive to Wnt signaling activity. Finally, a conspicuous apical tuft forms within a central *six3-*free territory within the apical plate. These similarities are most parsimoniously explained by common origin. We accordingly propose that the last common ancestor of bilaterians and cnidarians developed via primary larvae that possessed an apical tuft as part of a simple apical organ. A basal plexus may have formed directly underneath the apical organ, which was innervated by sensory-neurosecretory apical plate cells. We hypothesize that an ancient function of the apical organ was the control of metamorphosis and opsin-based ambient light perception. Various types of additional apical plate cells would then have subsequently been recruited to form part of the apical organ in the divergent bilaterian lineages. Our findings support an ancient and common origin of primary ciliated larvae.

## Methods

### Isolation of *Platynereis* genes and sequence analysis

Partial sequences for pdu-fgfr, pdu-foxq2, pdu-irx, pdu-foxJ1, pdu-hnf6, pdu-wnt4, pdu-frizzled5/8, pdu-sfrp1/5 and pdu-peropsin were assembled from *Platynereis* transcriptome and genome resources, amplified with specific primers, and extended with rapid amplification of cDNA ends PCR. Pdu-CTBL1, Pdu-bZIP-TF, Pdu-tektin-2, Pdu-spondin, Pdu-gbrl, Pdu-cpe, Pdu-smad2/3 and Pdu-klf2/4 were characterized during a whole mount *in situ* hybridization screen from expressed sequence tag clones (RT and DA, unpublished). The GenBank accession numbers for peropsin, foxj, fezf, onecut, fgfR, noggin, foxq2, frizzled4, frizzled5/8, frizzled9/10 and sfrp1/5 are [GenBank:KF844232] to [GenBank:KF844242] respectively. Accession numbers for ctbl1, spondin, gbr1, cpe, ces2 and klf are [GenBank:KF835846] to [GenBank:KF835851] respectively.

### *In situ* hybridizations and immunostainings

For *in situ* hybridizations at early stages, *Platynereis* larvae were fixed in 4% PFA, 0.1 M MOPS, 2 mM EGTA, 1 μM MgSO4 and 0.1% Tween-20, for 4 to 6 hours at 4°C, then rinsed in PTW and ice cold methanol, followed by storage in methanol at -20C.

*In situ* hybridization for early *Platynereis* larvae were performed as in [118,119] with the following modifications. Embryos were digested with 0.1 mg/mL proteinaseK for 30 seconds (Merck 7066304, Darmstadt, Germany). Following hybridization, 0.5 × SSC washes were replaced by 0.15 × SSC washes for 15 rather than 30 minutes. *In situ* hybridizations were performed for 48 hpf *Platynereis* and microRNA as previously published [22,118]. The axonal scaffold was counterstained with an antibody against tyrosinated or acetylated-tubulin (1:500, Sigma T9028 and T6793). Immunostainings were performed as described [12] with the following primary antibodies: mouse anti-acetylated tubulin (1:500, Sigma T6793), rabbit anti-serotonin (1:500, ImmunoStar 20080) and rabbit anti-FMRF (1:200, Phoenix Pharmaceuticals, H-047-29).

For the staining of mitotic cells, *Platynereis* larvae were incubated in 10 μM EdU (Click-iT EdU Imaging Kit, Invitrogen, C10340) from 22 to 24 hpf; EdU incorporation was detected after the incubation with secondary antibodies, following manufacturer instructions.

### Alsterpaullone and azakenpaullone treatments

*Platynereis* larvae were incubated from 12 to 24 hpf in five different concentrations of azakenpaullone (0.1 to 10 μM) in 0.5% dimethyl sulfoxide (DMSO) in filtered seawater. The number of embryos displaying wild-type, reduced or expanded expression patterns for episphere molecular markers were assessed from two different biological replicates (numbers of embryos displaying each pattern are indicated in Figure 5). As most genes assayed were significantly affected at 1 and 5 μM concentrations, we conducted washout experiments at these concentrations. Larvae were exposed to azakenpaullone from 12 to 24 hpf and subsequently washed out of the pharmacological treatment into 0.5% DMSO and incubated in 0.5% DMSO from 24 to 30 hpf. Controls were maintained in 0.5% DMSO from 12 to 24 hpf and washed into a new 0.5% DMSO treatment from 24 to 30 hpf. Embryos were examined and assessed in relation to control-treated embryos. Effects of alsterpaullone were also assayed, compared to those of azakenpaullone, and found to be similar to azakenpaullone treatments (Additional file 1: Figure S2).

### Microscopy and image processing

We used reflection microscopy [120] to acquire confocal images of *in situ* hybridization stainings. Fluorescent signals in Figure 2Q’ , Figure 4C and Figure 4C’ were obtained using the fluorescent signal emitted from NBT/BCIP precipitate, an alternate method to reflection microscopy. Confocal stacks were taken on a Leica TCS SPE with a 40× oil immersion objective. Images were processed with ImageJ, utilizing either brightest point or average intensity settings to generate projections. Subsequently, images were cropped and processed in Photoshop, Adobe, San Jose, California, USA; brightness and contrast were adjusted equally across the entire image.

For PrImR, average expression patterns at 48 hpf were obtained after image registration of *in situ* confocal scans on a common reference axonal scaffold, as described in [47].

### Molecular fingerprint analysis

Morphologically distinct apical organ cell types were identified by analyzing immunostained larvae at early stages (24 and 30 hpf).These cells were then located in 48 hpf larvae for the gene expression analysis with PrImR. The co-localization between two average gene expression patterns was inspected and visualized using the ‘Colocalization highlighter’ plugin in ImageJ.

Whenever a PrImR average expression pattern was not available for the gene and/or the stage of interest, specimens stained with the gene of interest and tyrosinated tubulin were inspected under fluorescence microscopy.

Hierarchical clustering of molecular fingerprints was carried in R (the R Project for Statistical Computing) from the dataset in Additional file 1: Figure S5, using Pearson correlation and average linkage.

### Phylogenetic analyses for gene orthologies

*Platynereis dumerilii* gene coding sequences used in this study were isolated as described above. Sequence data from the lophotrochozoans *Lottia gigantea* and *Capitella teleta* and the cnidarian *Nematostella vectensis* were identified on their respective JGI genome portal webservers. Additional sequences used in the analyses were downloaded from Treefam [121]. Multiple alignments of predicted proteins were generated with MUSCLE using the default settings [122] and were subsequently inspected and corrected by eye. Full alignments were trimmed using G-blocks [123] and were run through ProtTest using the default settings to determine the optimum evolutionary model for phylogenetic analyses [124]. Neighbor joining trees were constructed using MEGA [125] and maximum likelihood analyses were conducted using PhyML [126] with the amino acid substitution models specified from ProtTest. Phylogenetic trees are available in Additional file 2.

## Abbreviations

DMSO: dimethyl sulfoxide; Fgf: fibroblast growth factor; hpf: Hours post-fertilization; PCR: polymerase chain reaction; PrImR: profiling by image registration; Tgf: transforming growth factor.

## Competing interests

The authors declare that they have no competing interests.

## Authors’ contributions

HM and DA designed the study. HM collected *in situ* expression data and performed alsterpaullone and azakenpaullone treatments. MAT generated and analyzed molecular fingerprint data, performed clustering analysis and characterized cell morphologies. RT cloned multiple genes and generated averaged expression data for Profiling by Image Registration. PRS cloned the fgfR gene. AL contributed to TrpV cloning and expression analysis. TL generated phylogenetic analyses for gene orthologies. HM, MAT and DA wrote the paper. All authors read and approved the final manuscript.

## Supplementary Material

Additional file 1: Figure S1 miR183 expression in a 48 hpf larva. **Figure S2.** Alsterpaullone treated embryos. **Figure S3.** Azakenpaullone treated embryos following washout. **Figure S4.** Percentages of affected embryos in azakenpaullone treatments. **Figure S5.** PrImR analysis of the expression of transcription factors, miRNAs and differentiation markers in defined apical organ cell types. Click here for file

Additional file 2 Phylogenetic analysis for the assignment of gene orthology. Click here for file
