# Supplementary material for: Larval body patterning and apical organs are conserved in animal evolution
Source: BMC Biol. 2014 Jan 29;12:7. doi: 10.1186/1741-7007-12-7 (PMC3939940; doi:10.1186/1741-7007-12-7)
Supplement: Additional file 1: Figure S1 — miR183 expression in a 48 hpf larva. Figure S2. Alsterpaullone treated embryos. Figure S3. Azakenpaullone treated embryos following washout. Figure S4. Percentages of affected embryos in azakenpaullone treatments. Figure S5. PrImR analysis of the expression of transcription factors, miRNAs and differentiation markers in defined apical organ cell types. [file 1741-7007-12-7-S1.doc]

**
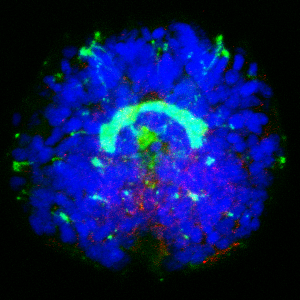
**

**Figure S1 miR183 expression in a 48hpf larva.**

miR183 is visible in a patch of ventral apical organ cells at 48hpf.

**Figure S2 Alsterpaullone treated embryos**

The pharmacological agent alsterpaullone regulates apical plate gene expression in *Platynereis* larvae. Embryos in panels A-C were treated from 9 to 24hpf while those in panels D-G were treated from 18 to 24hpf. A. FgfR expression is expanded in the apical plate in embryos treated from 18-24hpf while Otp (B) and Six3 (C) expression are reduced or absent. D. Punctate expression of *Irx* associated with apical organ cells is lost after treatment with alsterpaullone. E. *FoxQ2* expression is lost in all but the most apical cells of the epishere in alsterpaullone treatments. G. Punctate expression of *otp* is reduced or absent in alsterpaullone treatments. F *Six3* is absent from the apical plate after alsterpaullone treatment. Arrowheads point to small populations of cells expressing the genes indicated.

**Figure S3 Azakenpaullone treated embryos following washout**

Embryos treated with DMSO only (A-E) or with azakenpaullone (A’’-E’’) from 12-24hpf. Embryos treated from 12-24hpf in DMSO and washed into DMSO as washout controls from 24-30hpf (A’-E’) and those treated from 12-24hpf with azakenpaullone and then washed into DMSO from 24-30hpf (A’’-E’’).


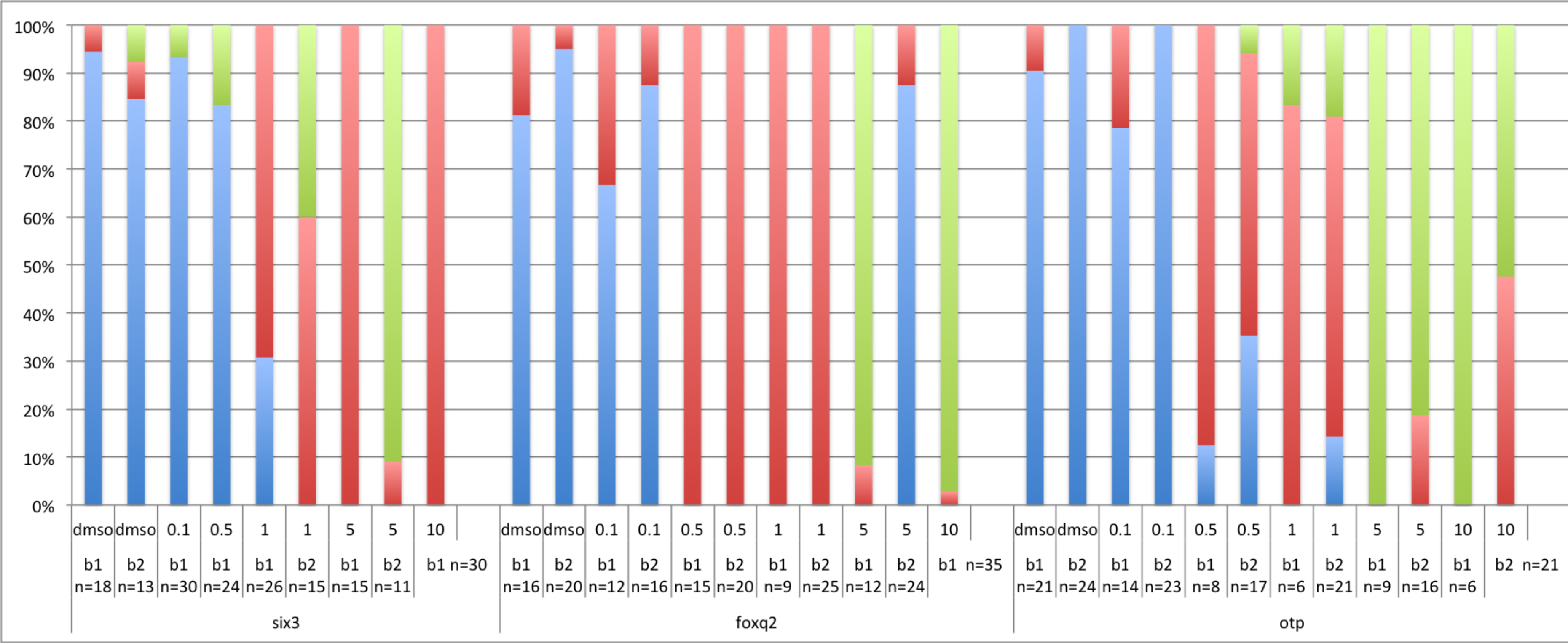


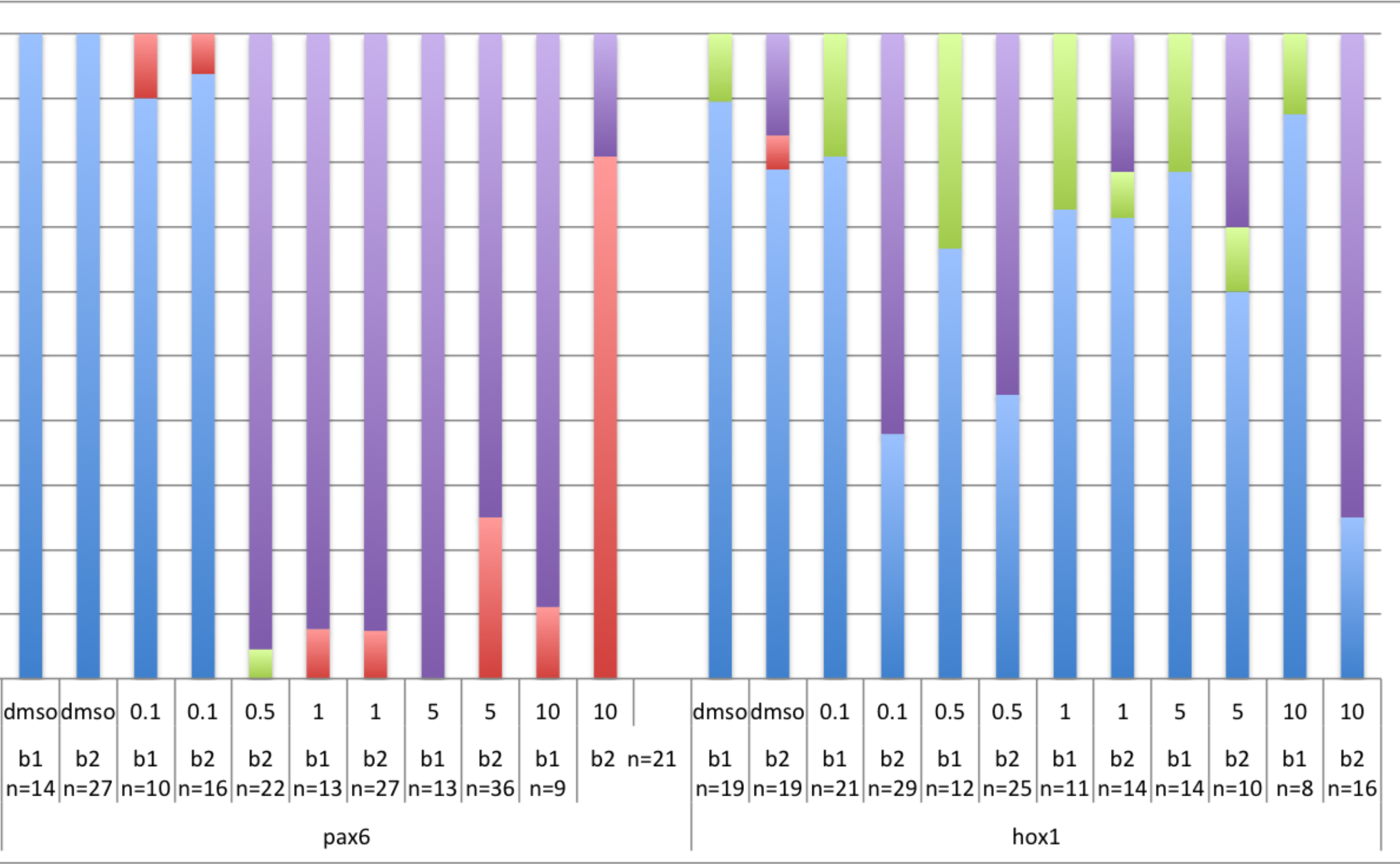

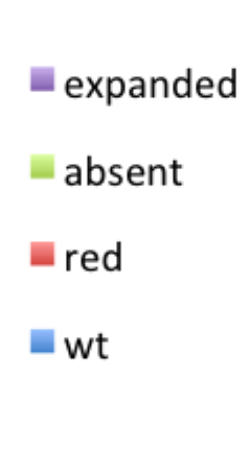


**Figure S4 Percentages of affected embryos in azakenpaullone treatments**

Percentages of embryos for each of two biological replicates at 0, 1, and 5 μM concentrations of azakenpaullone showing wildtype, reduced, expanded or absent expression domains for the transcripts indicated. The number of embryos for each treatment is indicated. An “expanded” expression pattern for Hox1 (as seen in biological replicate two) indicates that very faint expression outside the two ampullary expressing cells was also detected.

**Figure S5 PrImR analysis of the expression of transcription factors, miRNAs and differentiation markers in defined apical organ cell types.**

For each set, the first image represents the cell(s) of interest (yellow, extrapolated from average gene expression data) in relationship to the reference axonal scaffold (blue). All the other images show the coexpression of restricted cell-type markers, where white marks colocalizing pixels.

A. Apical organ serotonergic cell, demarcated by the expression of lmx1ab (green), Z projections of slice numbers 7 to 22, i.e., 15 μm thick in the reference scaffold image stack (cfr Tomer et al 2010). B. DLamide+ medial cell, demarcated by the expression of DLamide (green), Z projections of slice numbers 9 to 16; C. WLDamide+ cell, demarcated by the expression of WLDamide (green), Z projections of slice numbers 11 to 18; D. Klf2/4+ cell, demarcated by the expression of Klf2/4 (green), Z projections of slice numbers 10 to 20; E. FMRFamide+ cell, demarcated by the expression of FMRFamide (green), Z projections of slice numbers 7 to 16; F. ampullary cells, demarcated by the expression of miR-34 (green), Z projections of slice numbers 10 to 20; G. crescent cells, demarcated by the expression of tubby (green), Z projections of slice numbers 7 to 11; H. putative chemosensory cells, demarcated by the expression of tektin-2 (green), Z projections of slice numbers 10 to 22;I. prototroch cells, demarcated by the expression of tektin-2 (green), Z projections of slice numbers 55 to 75.

Beer, A. J., C. Moss, et al. (2001). "Development of serotonin-like and SALMFamide-like immunoreactivity in the nervous system of the sea urchin Psammechinus miliaris." The Biological Bulletin **200**(3): 268.

Bertrand, S., A. Camasses, et al. (2011). "Amphioxus FGF signaling predicts the acquisition of vertebrate morphological traits." Proceedings of the National Academy of Sciences **108**(22): 9160.

Burke, R., L. Angerer, et al. (2006). "A genomic view of the sea urchin nervous system." Dev Biol **300**(1): 434-460.

Candiani, S., L. Moronti, et al. (2011). "A study of neural-related microRNAs in the developing amphioxus." EvoDevo **2**(1): 15.

Chevalier, S., A. Martin, et al. (2006). "Polarised expression of FoxB and FoxQ2 genes during development of the hydrozoan Clytia hemisphaerica." Development Genes and Evolution **216**(11): 709-720.

Croce, J., L. Duloquin, et al. (2006). "Frizzled5/8 is required in secondary mesenchyme cells to initiate archenteron invagination during sea urchin development." Development **133**(3): 547-557.

Darras, S., J. Gerhart, et al. (2011). "β-Catenin specifies the endomesoderm and defines the posterior organizer of the hemichordate Saccoglossus kowalevskii." Development **138**(5): 959.

Di Bernardo, M., S. Castagnetti, et al. (1999). "Spatially restricted expression of PlOtp, a Paracentrotus lividus orthopedia-related homeobox gene, is correlated with oral ectodermal patterning and skeletal morphogenesis in late-cleavage sea urchin embryos." Development **126**(10): 2171.

Dunn, E. F., V. N. Moy, et al. (2007). "Molecular paleoecology: using gene regulatory analysis to address the origins of complex life cycles in the late Precambrian." Evolution & Development **9**(1): 10-24.

Hejnol, A. and M. Q. Martindale (2008). "Acoel development indicates the independent evolution of the bilaterian mouth and anus." Nature **456**(7220): 382-386.

Holland, N. D. and L. Z. Holland (1993). "Serotonin-containing Cells in the Nervous System and Other Tissues During Ontogeny of a Lancelet, Branchiostoma floridae." Acta Zoologica **74**(3): 195-204.

Holland, N. D., G. Panganiban, et al. (1996). "Sequence and developmental expression of AmphiDll, an amphioxus Distal-less gene transcribed in the ectoderm, epidermis and nervous system: insights into evolution of craniate forebrain and neural crest." Development **122**(9): 2911-2920.

Howard-Ashby, M., S. C. Materna, et al. (2006). "Identification and characterization of homeobox transcription factor genes in S. purpuratus, and their expression in embryonic development." Dev Biol **doi: 10.1016/j.ydbio.2006.08.039**.

Illies, M. R., M. T. Peeler, et al. (2002). "Cloning and developmental expression of a novel, secreted frizzled-related protein from the sea urchin, Strongylocentrotus purpuratus." Mechanisms of development **113**(1): 61-64.

Irimia, M., C. Pi√±eiro, et al. (2010). "Conserved developmental expression of Fezf in chordates and Drosophila and the origin of the Zona Limitans Intrathalamica (ZLI) brain organizer." EvoDevo **1**(1): 7.

Kozmik, Z., N. D. Holland, et al. (2007). "Pax-Six-Eya-Dach network during amphioxus development: conservation in vitro but context specificity in vivo." Dev Biol **306**(1): 143-159.

Kumburegama, S., N. Wijesena, et al. (2011). "Strabismus-mediated primary archenteron invagination is uncoupled from Wnt/β-catenin-dependent endoderm cell fate specification in Nematostella vectensis (Anthozoa, Cnidaria): Implications for the evolution of gastrulation." EvoDevo **2**(1): 1-15.

Kusserow, A., K. Pang, et al. (2005). "Unexpected complexity of the Wnt gene family in a sea anemone." Nature **433**(7022): 156-160.

Lacalli, T. C. (1996). "Frontal eye circuitry, rostral sensory pathways and brain organization in amphioxus larvae: evidence from 3D reconstructions." Philosophical Transactions: Biological Sciences: 243-263.

Lacalli, T. C. and S. J. Kelly (2000). "The infundibular balance organ in amphioxus larvae and related aspects of cerebral vesicle organization." Acta Zoologica **81**(1): 37-47.

Lee, P. N., K. Pang, et al. (2006). A WNT of things to come: evolution of Wnt signaling and polarity in cnidarians, Elsevier.

Lowe, C. J., M. Wu, et al. (2003). "Anteroposterior patterning in hemichordates and the origins of the chordate nervous system." Cell **113**: 853-865.

Marlow, H. Q. (2011). Cell type diversity and developmental mechanisms in larval and adult anthozoan cnidarians, University of Hawai'i at Manoa.

Marlow, H. Q., M. Srivastava, et al. (2009). "Anatomy and development of the nervous system of Nematostella vectensis, an anthozoan cnidarian." Dev Neurobiol **69**(4): 235-254.

Matus, D. Q., K. Pang, et al. (2006). "Deep evolutionary roots for bilaterality in the metazoa." Proc Natl Acad Sci U S A.

Matus, D. Q., G. H. Thomsen, et al. (2007). "FGF signaling in gastrulation and neural development in Nematostella vectensis, an anthozoan cnidarian." Dev Genes Evol **217**(2): 137-148.

Mazza, M. E., K. Pang, et al. (2007). "Genomic organization, gene structure, and developmental expression of three clustered otx genes in the sea anemone Nematostella vectensis." Journal of Experimental Zoology Part B: Molecular and Developmental Evolution **308**(4): 494-506.

Mazza, M. E., K. Pang, et al. (2010). "A conserved cluster of three PRD-class homeobox genes (homeobrain, rx and orthopedia) in the Cnidaria and Protostomia."

Mitsunaga-Nakatsubo, K., K. Akasaka, et al. (1998). "Differential expression of sea urchin Otx isoform (HpOtx~ E and HpOtx~ L) mRNAs during early development." INTERNATIONAL JOURNAL OF DEVELOPMENTAL BIOLOGY **42**: 645-651.

Miyamoto, N., Y. Nakajima, et al. (2010). "Development of the nervous system in the acorn worm Balanoglossus simodensis: insights into nervous system evolution." Evolution & Development **12**(4): 416-424.

Nakajima, Y., T. Humphreys, et al. (2004). "Development and neural organization of the tornaria larva of the Hawaiian hemichordate, Ptychodera flava." Zoological science **21**(1): 69-78.

Nederbragt, A. J., P. te Welscher, et al. (2002). "Novel and conserved roles for orthodenticle/otx and orthopedia/otp orthologs in the gastropod mollusc Patella vulgata." Development genes and evolution **212**(7): 330-337.

Nezlin, L. P. and V. V. Yushin (2004). "Structure of the nervous system in the tornaria larva of Balanoglossus proterogonius (Hemichordata: Enteropneusta) and its phylogenetic implications." Zoomorphology **123**(1): 1-13.

Ogasawara, M. (2000). "Overlapping expression of amphioxus homologs of the thyroid transcription factor-1 gene and thyroid peroxidase gene in the endostyle: insight into evolution of the thyroid gland." Dev Genes Evol **210**(5): 231-242.

Onai, T., H. C. Lin, et al. (2009). "Retinoic acid and Wnt/β-catenin have complementary roles in anterior/posterior patterning embryos of the basal chordate amphioxus." Dev Biol **332**(2): 223-233.

Pierce, M. L., M. D. Weston, et al. (2008). "MicroRNA-183 family conservation and ciliated neurosensory organ expression." Evolution & development **10**(1): 106-113.

Poustka, A. J., A. K√ºhn, et al. (2007). "A global view of gene expression in lithium and zinc treated sea urchin embryos: new components of gene regulatory networks." Genome biology **8**(5): R85.

Rentzsch, F., J. H. Fritzenwanker, et al. (2008). "FGF signalling controls formation of the apical sensory organ in the cnidarian Nematostella vectensis." Development **135**(10): 1761-1769.

Reuter, M., O. Raikova, et al. (1998). "An endocrine brain? The pattern of FMRF-amide immunoreactivity in Acoela (Plathelminthes)." Tissue and Cell **30**(1): 57-63.

Reuter, M., O. Raikova, et al. (2001). "Organisation of the nervous system in the Acoela: an immunocytochemical study." Tissue and Cell **33**(2): 119-128.

Röttinger, E. and M. Q. Martindale (2011). "Ventralization of an indirect developing hemichordate by NiCl2 suggests a conserved mechanism of dorso-ventral (D/V) patterning in Ambulacraria (Hemichordates & Echinoderms)." Developmental Biology.

Ryan, J. F., M. E. Mazza, et al. (2007). "Pre-bilaterian origins of the Hox cluster and the Hox code: evidence from the sea anemone, Nematostella vectensis." PLoS ONE **2**(1): e153.

Scholpp, S. and A. Lumsden (2010). "Building a bridal chamber: development of the thalamus." Trends in neurosciences **33**(8): 373-380.

Schubert, M., L. Z. Holland, et al. (2000). "Characterization of two amphioxus Wnt genes (AmphiWnt4 and AmphiWnt7b) with early expression in the developing central nervous system." Developmental Dynamics **217**(2): 205-215.

Tagawa, K., N. Satoh, et al. (2001). "Molecular studies of hemichordate development: a key to understanding the evolution of bilateral animals and chordates." Evolution & Development **3**(6): 443-454.

Takacs, C. M., G. Amore, et al. (2004). "Expression of an NK2 homeodomain gene in the apical ectoderm defines a new territory in the early sea urchin embryo." Developmental biology **269**(1): 152-164.

Takacs, C. M., V. N. Moy, et al. (2002). "Testing putative hemichordate homologues of the chordate dorsal nervous system and endostyle: expression of NK2. 1 (TTF-1) in the acorn worm Ptychodera flava (Hemichordata, Ptychoderidae)." Evolution & Development **4**(6): 405-417.

Tu, Q., C. T. Brown, et al. (2006). "Sea urchin Forkhead gene family: phylogeny and embryonic expression." Developmental biology **300**(1): 49-62.

Williams, N. A. and P. W. H. Holland (1996). "Old head on young shoulders." Nature **383**: 490.

Yaguchi, S., J. Yaguchi, et al. (2008). "A Wnt-FoxQ2-nodal pathway links primary and secondary axis specification in sea urchin embryos." Dev Cell **14**(1): 97-107.

Yaguchi, S., J. Yaguchi, et al. (2011). "Fez function is required to maintain the size of the animal plate in the sea urchin embryo." Development **138**(19): 4233-4243.

Yankura, K., M. Martik, et al. "Uncoupling of complex regulatory patterning during evolution of larval development in echinoderms." BMC biology **8**(1): 143.

Yu, J. K., F. Mazet, et al. (2008). "The Fox genes of Branchiostoma floridae." Dev Genes Evol **218**(11): 629-638.

Zhang, Y. and B. Mao (2010). "Embryonic expression and evolutionary analysis of the amphioxus Dickkopf and Kremen family genes." Journal of Genetics and Genomics **37**(9): 637-645.
