# Supplementary material for: Larval body patterning and apical organs are conserved in animal evolution
Source: BMC Biol. 2014 Jan 29;12:7. doi: 10.1186/1741-7007-12-7 (PMC3939940; doi:10.1186/1741-7007-12-7)
Supplement: Additional file 2 — Phylogenetic analysis for the assignment of gene orthology. [file 1741-7007-12-7-S2.pdf]

Ces-2 NJ

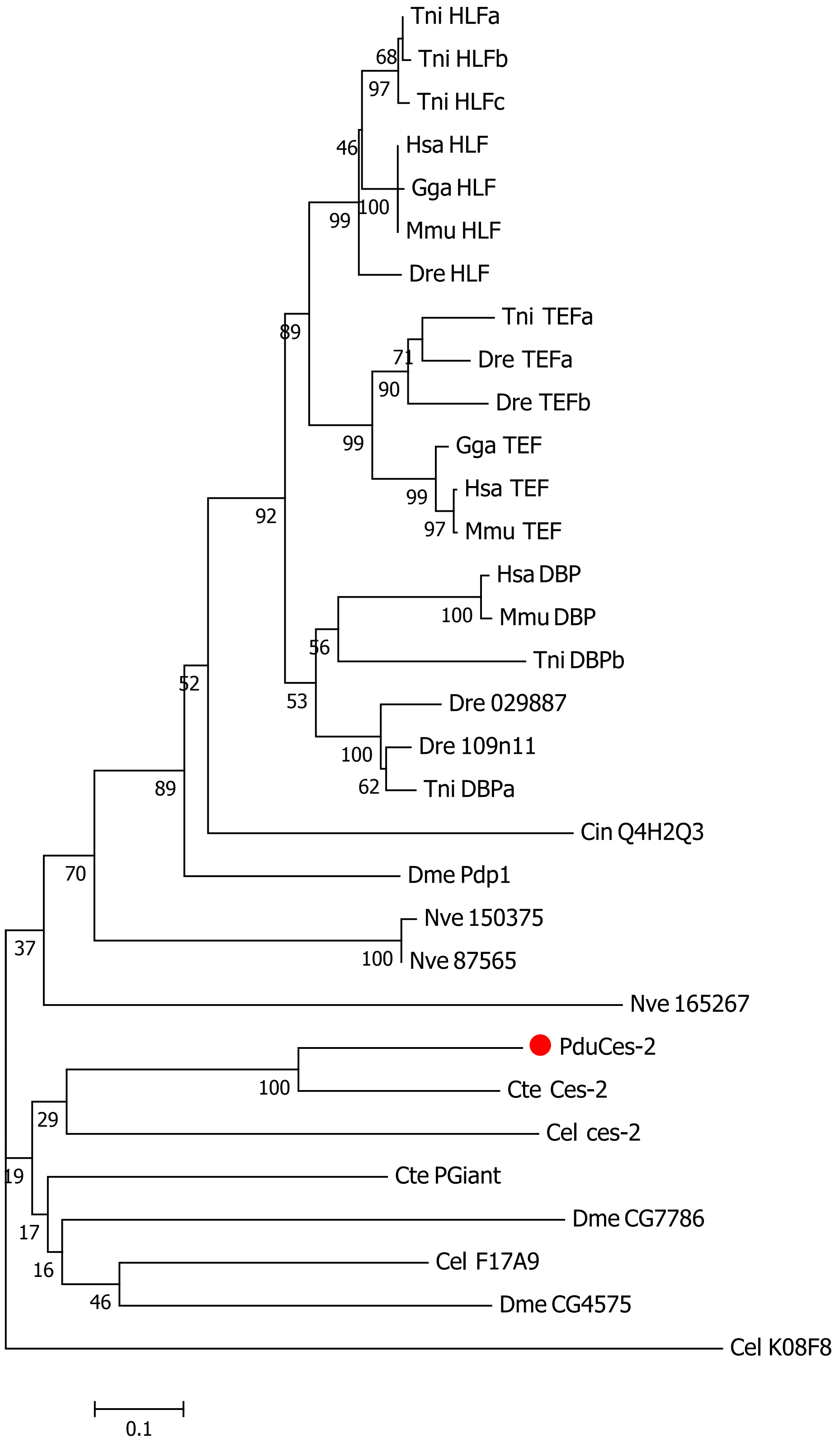
Tni HLFa
68
Tni HLFb
97
Tni HLFc
46
Hsa HLF
Gga HLF
100
99
Mmu HLF
Dre HLF
Tni TEFa
89
71
Dre TEFa
90
Dre TEFb
99
Gga TEF
Hsa TEF
99
97
Mmu TEF
92
Hsa DBP
100
Mmu DBP
56
Tni DBPb
52
53
Dre 029887
Dre 109n11
100
62
Tni DBPa
89
Cin Q4H2Q3
70
Dme Pdp1
Nve 150375
37
100
Nve 87565
Nve 165267
PduCes-2
100
Cte Ces-2
29
Cel ces-2
19
Cte PGiant
17
Dme CG7786
16
Cel F17A9
46
Dme CG4575
Cel K08F8
0.1

Ces-2 ML

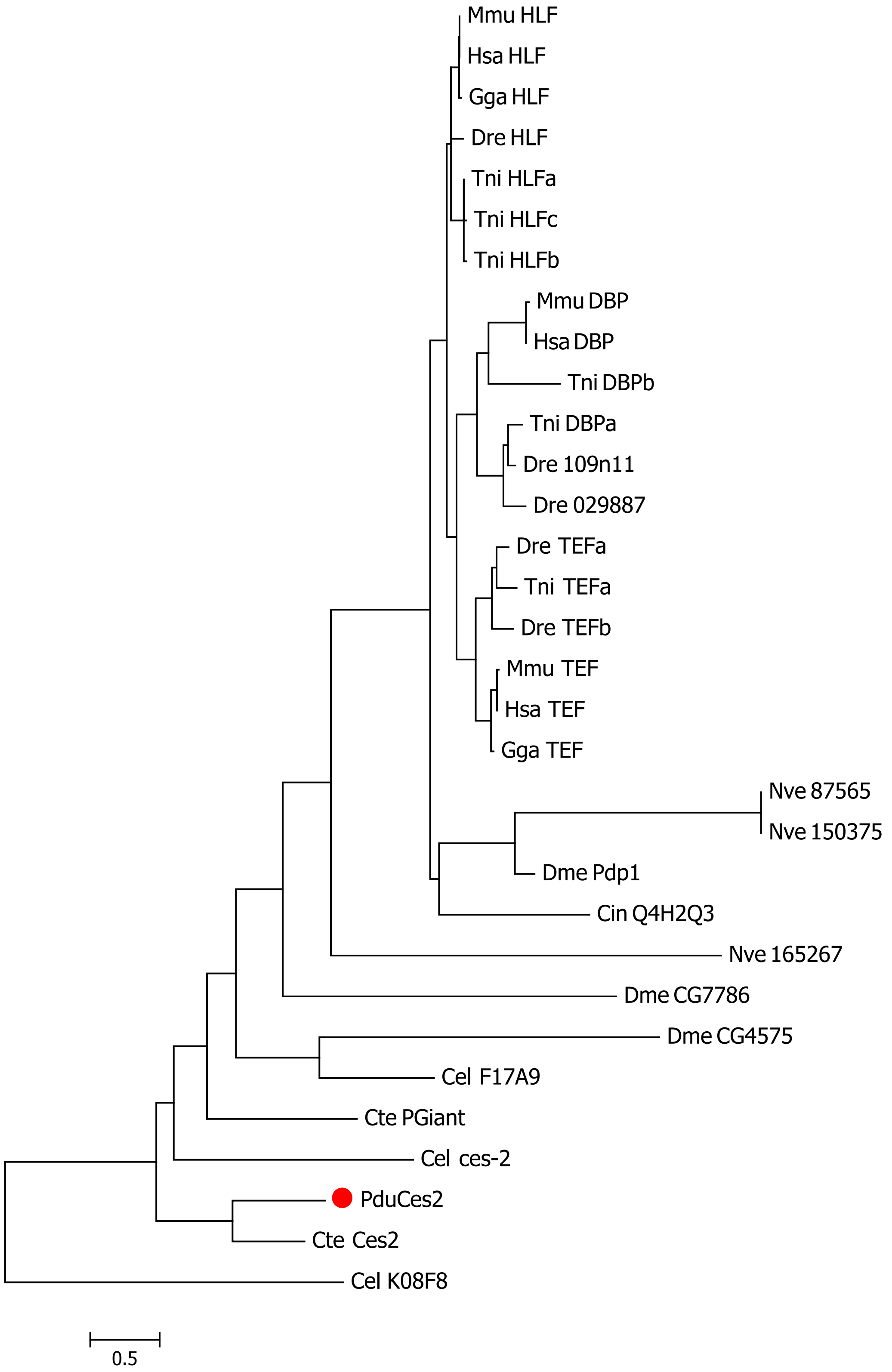

Mmu HLF
Hsa HLF
Gga HLF
Dre HLF
Tni HLFa
Tni HLFc
Tni HLFb
Mmu DBP
Hsa DBP
Tni DBPb
Tni DBPa
Dre 109n11
Dre 029887
Dre TEFa
Tni TEFa
Dre TEFb
Mmu TEF
Hsa TEF
Gga TEF
Nve 87565
Nve 150375
Dme Pdp1
Cin Q4H2Q3
Nve 165267
Dme CG7786
Dme CG4575
Cel F17A9
Cte PGiant
Cel ces-2
PduCes2
Cte Ces2
Cel K08F8
0.5

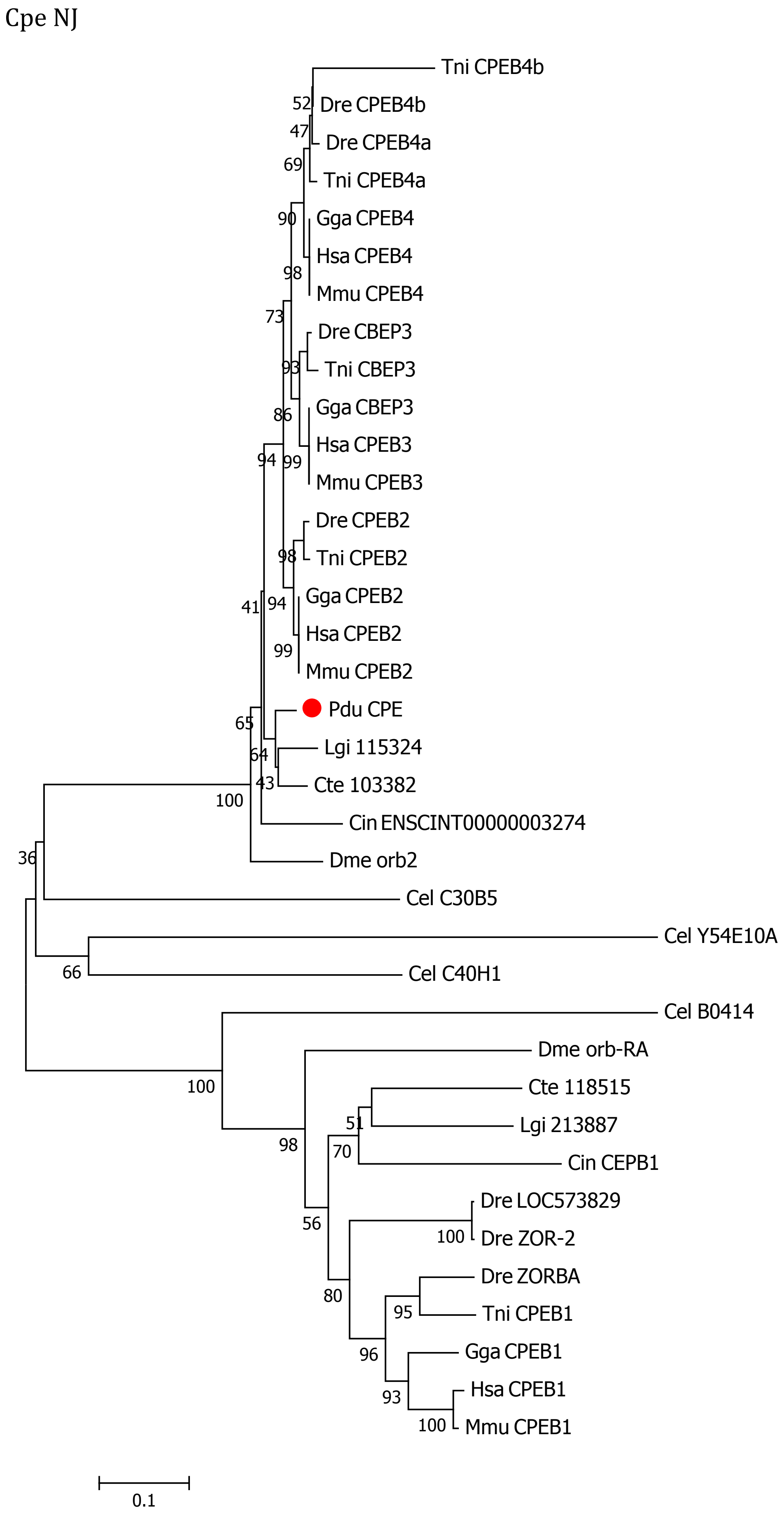
Cpe NJ
Tni CPEB4b
Dre CPEB4b
Dre CPEB4a
Tni CPEB4a
Gga CPEB4
Hsa CPEB4
Mmu CPEB4
Dre CBEP3
Tni CBEP3
Gga CBEP3
Hsa CPEB3
Mmu CPEB3
Dre CPEB2
Tni CPEB2
Gga CPEB2
Hsa CPEB2
Mmu CPEB2
Pdu CPE
Lgi 115324
Cte 103382
Cin ENSCINT00000003274
Dme orb2
Cel C30B5
Cel Y54E10A
Cel C40H1
Cel B0414
Dme orb-RA
Cte 118515
Lgi 213887
Cin CEPB1
Dre LOC573829
Dre ZOR-2
Dre ZORBA
Tni CPEB1
Gga CPEB1
Hsa CPEB1
Mmu CPEB1
52
47
69
90
98
73
93
86
94
99
98
41
94
99
65
64
43
100
36
66
100
51
98
70
56
100
80
95
96
93
100
0.1

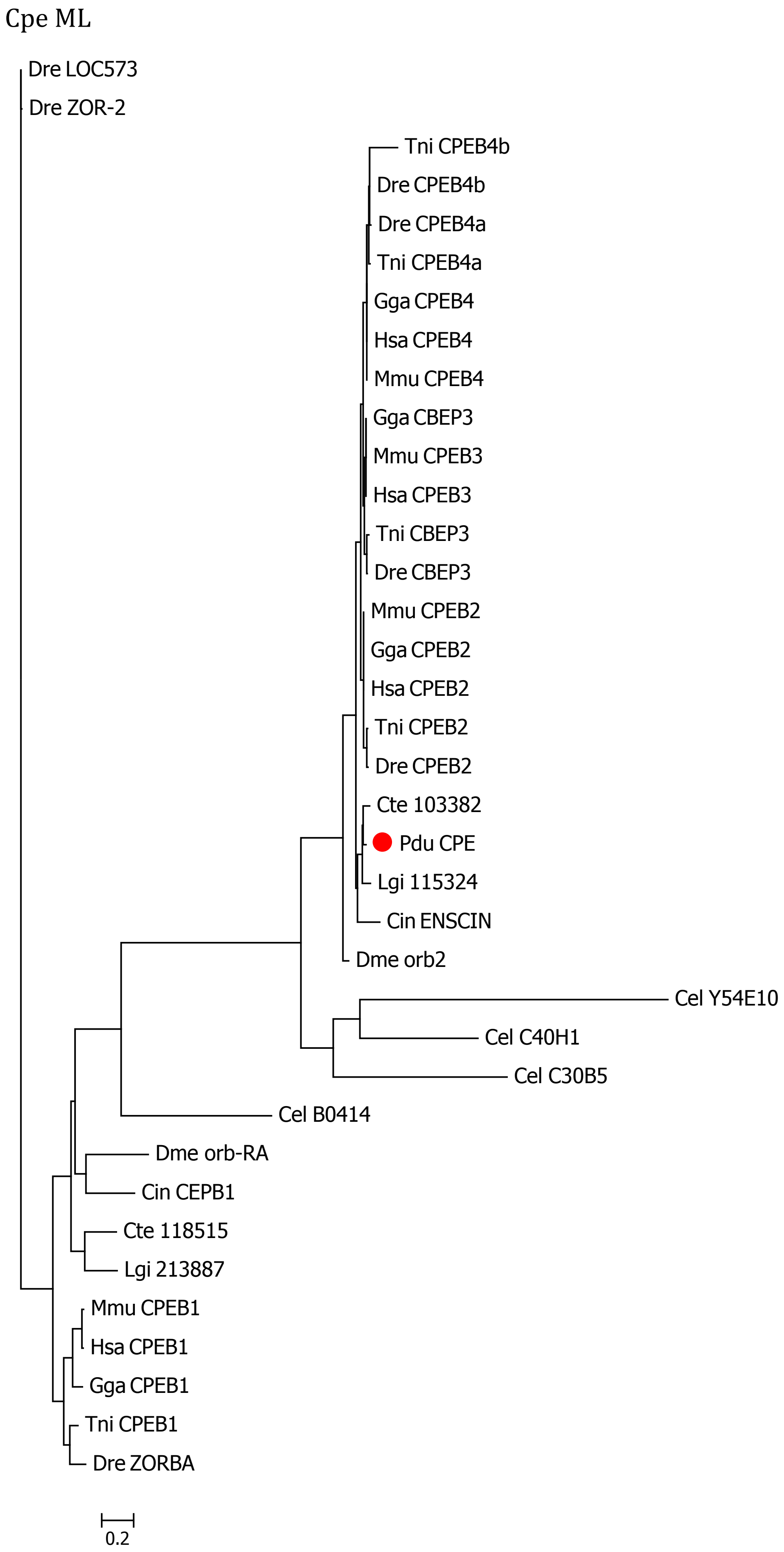
Cpe ML
Dre LOC573
Dre ZOR-2
Tni CPEB4b
Dre CPEB4b
Dre CPEB4a
Tni CPEB4a
Gga CPEB4
Hsa CPEB4
Mmu CPEB4
Gga CBEP3
Mmu CPEB3
Hsa CPEB3
Tni CBEP3
Dre CBEP3
Mmu CPEB2
Gga CPEB2
Hsa CPEB2
Tni CPEB2
Dre CPEB2
Cte 103382
Pdu CPE
Lgi 115324
Cin ENSCIN
Dme orb2
Cel Y54E10
Cel C40H1
Cel C30B5
Cel B0414
Dme orb-RA
Cin CEPB1
Cte 118515
Lgi 213887
Mmu CPEB1
Hsa CPEB1
Gga CPEB1
Tni CPEB1
Dre ZORBA
0.2

Ctbl1 NJ

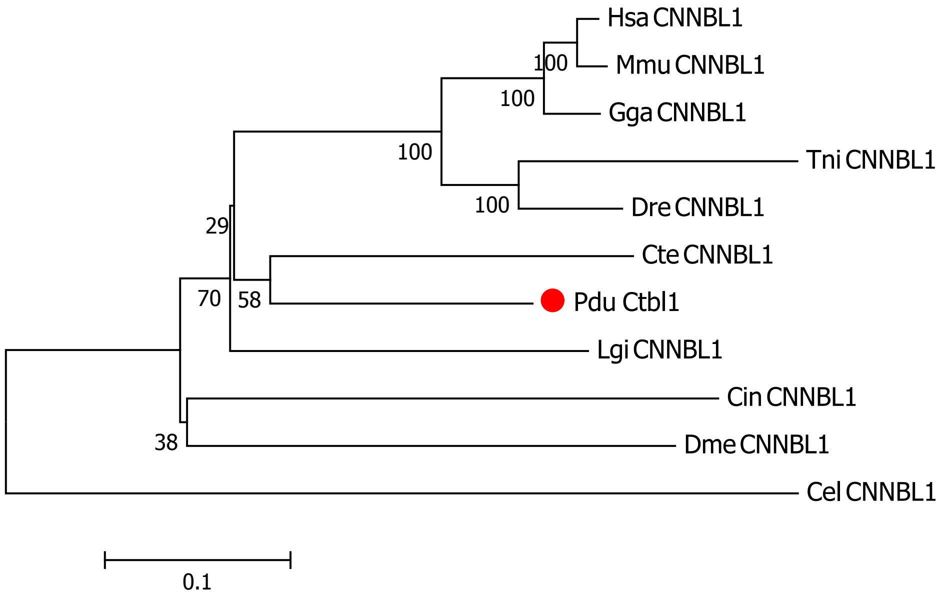


Ctbl1 ML

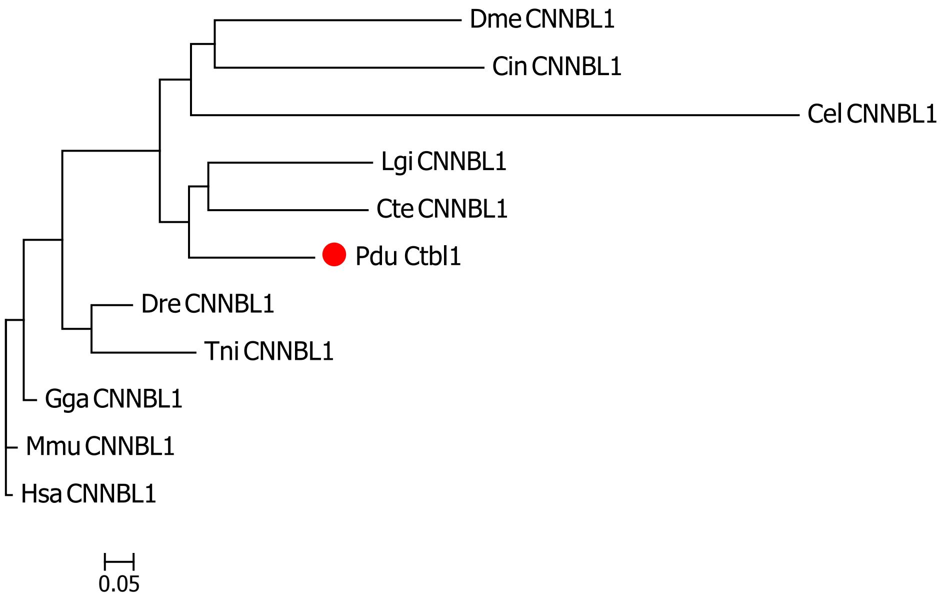

Fezf NJ

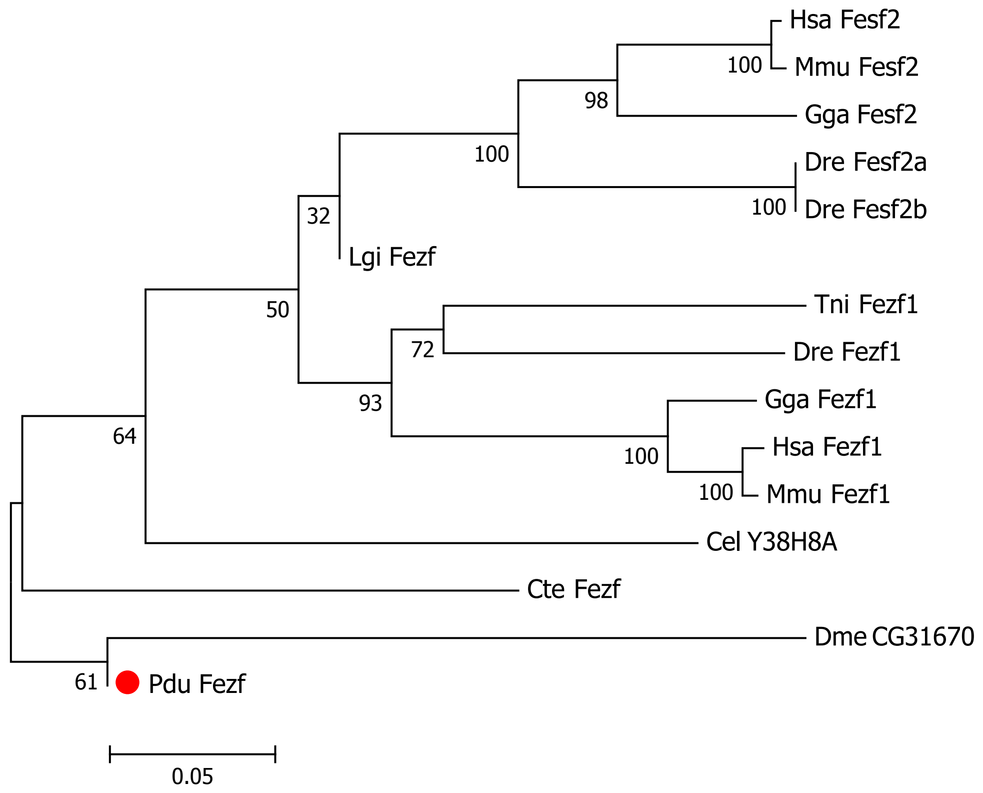


Fezf ML

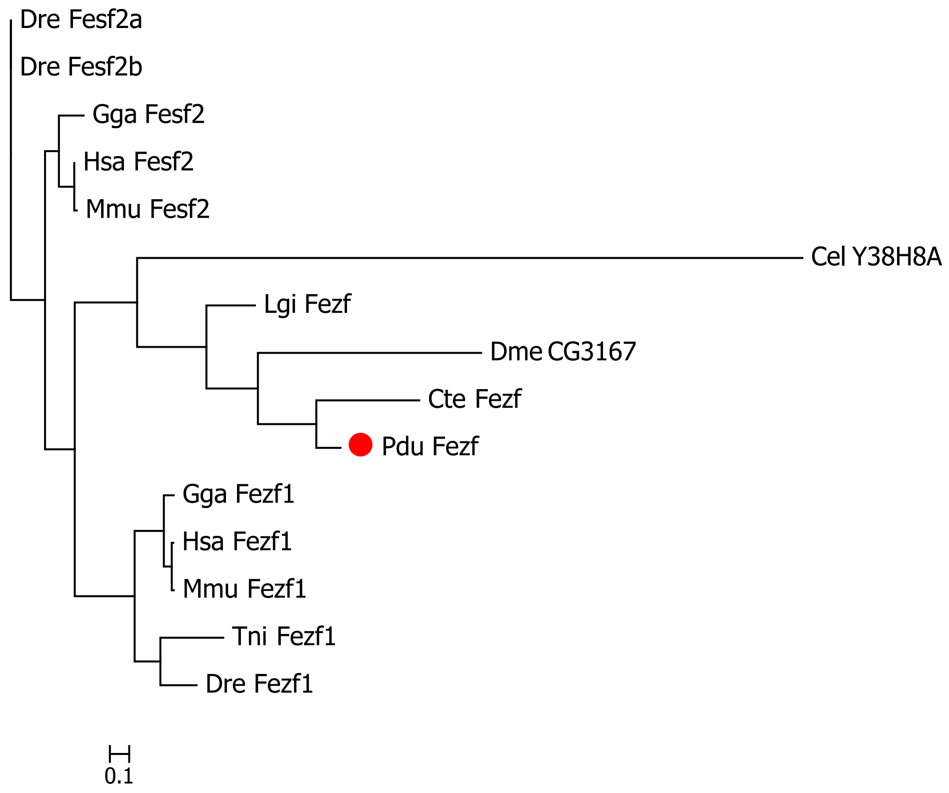

FoxJ NJ

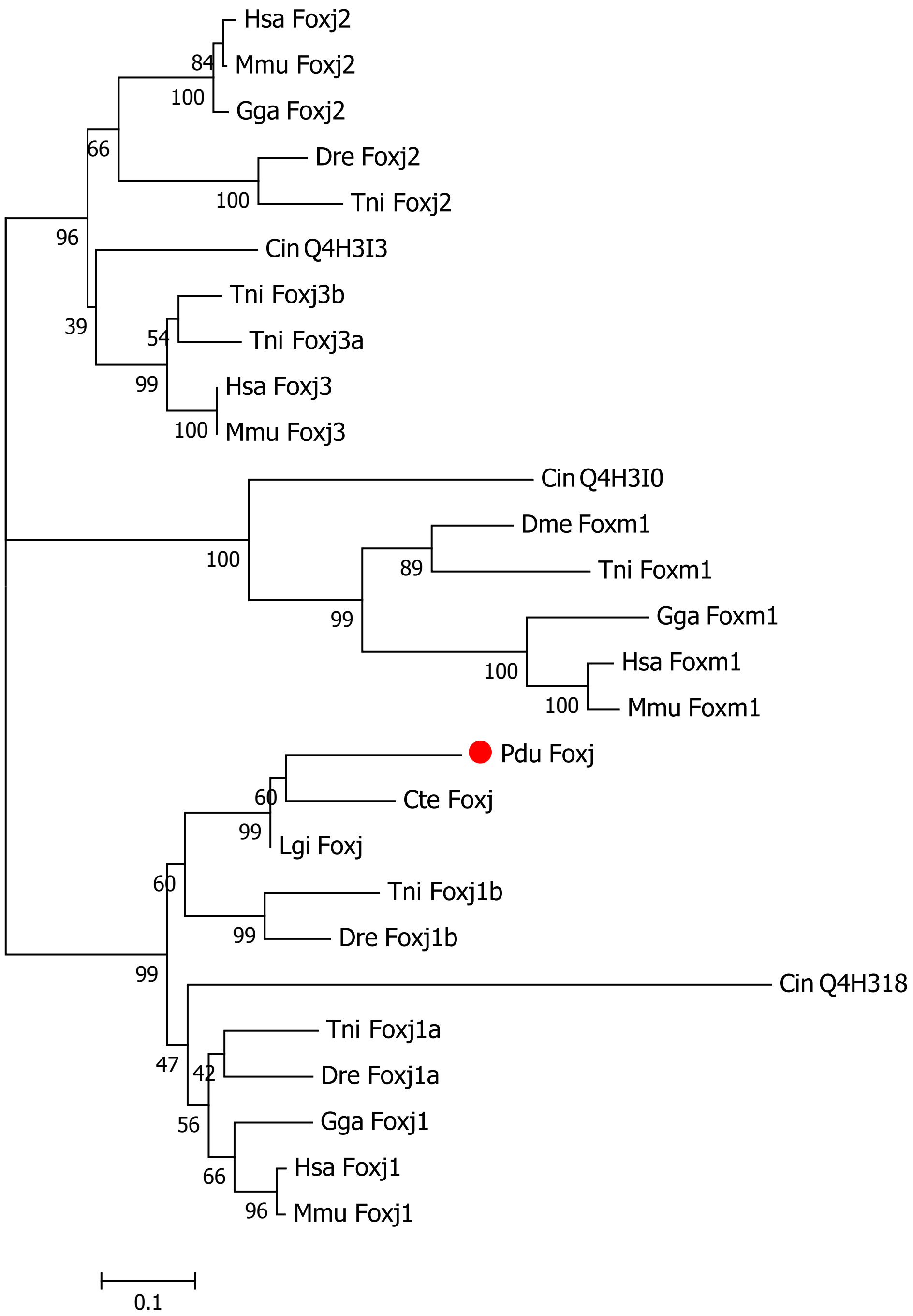
Hsa Foxj2
84
Mmu Foxj2
100
Gga Foxj2
66
Dre Foxj2
100
Tni Foxj2
96
Cin Q4H3I3
Tni Foxj3b
39
54
Tni Foxj3a
99
Hsa Foxj3
100
Mmu Foxj3
Cin Q4H3I0
Dme Foxm1
100
89
Tni Foxm1
99
Gga Foxm1
100
Hsa Foxm1
100
Mmu Foxm1
Pdu Foxj
60
Cte Foxj
99
Lgi Foxj
60
Tni Foxj1b
99
Dre Foxj1b
99
Cin Q4H318
Tni Foxj1a
47
42
Dre Foxj1a
56
Gga Foxj1
66
Hsa Foxj1
96
Mmu Foxj1
0.1

FoxJ ML

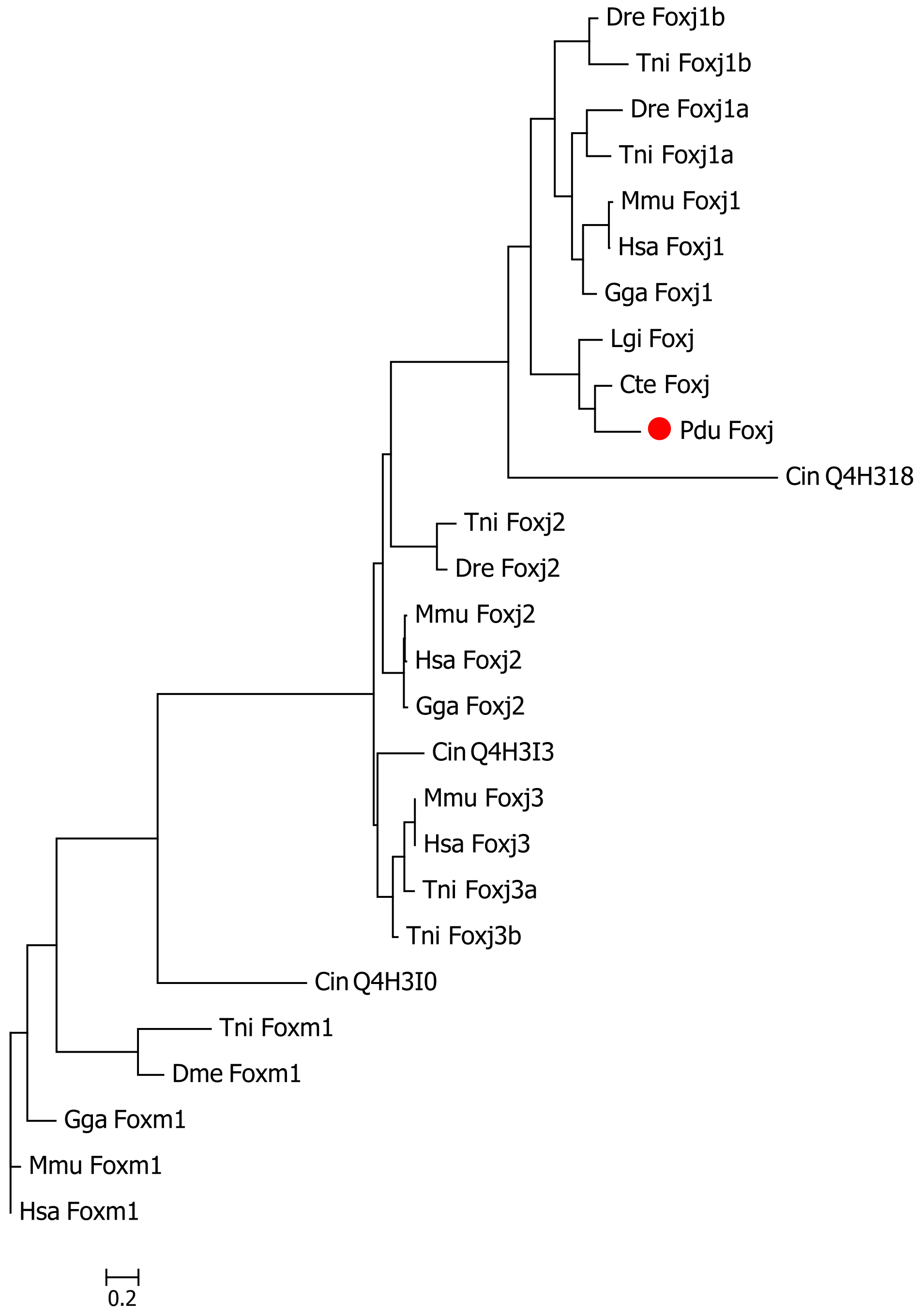
Dre Foxj1b
Tni Foxj1b
Dre Foxj1a
Tni Foxj1a
Mmu Foxj1
Hsa Foxj1
Gga Foxj1
Lgi Foxj
Cte Foxj
Pdu Foxj
Cin Q4H318
Tni Foxj2
Dre Foxj2
Mmu Foxj2
Hsa Foxj2
Gga Foxj2
Cin Q4H3I3
Mmu Foxj3
Hsa Foxj3
Tni Foxj3a
Tni Foxj3b
Cin Q4H3I0
Tni Foxm1
Dme Foxm1
Gga Foxm1
Mmu Foxm1
Hsa Foxm1
0.2

Frizzled NJ

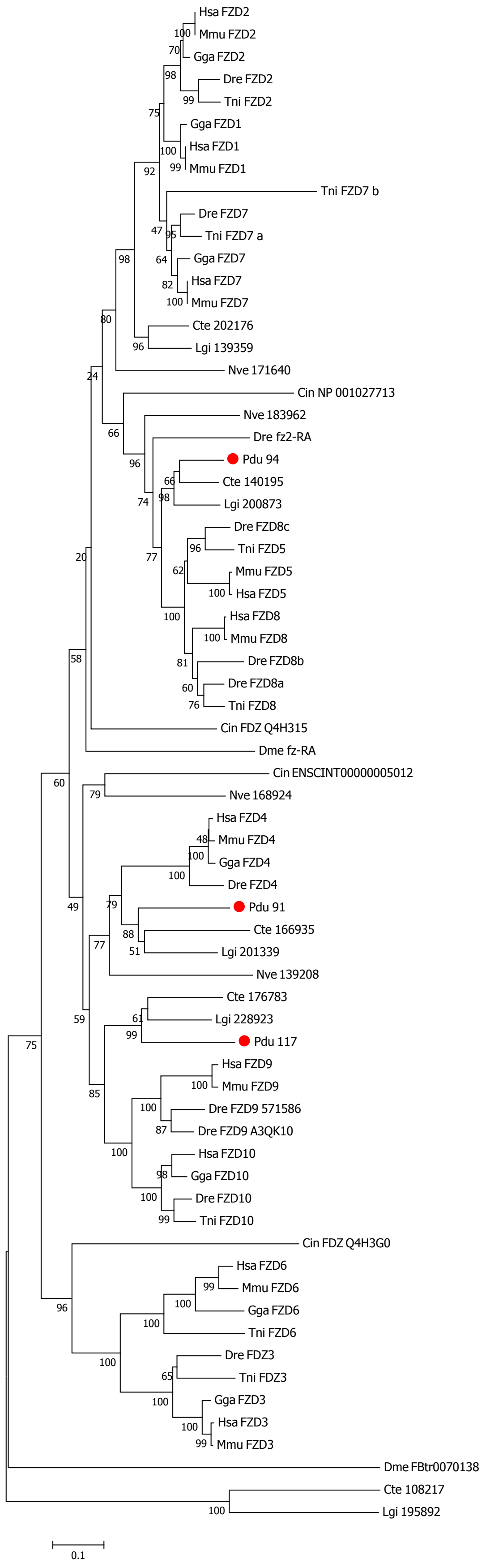
Hsa FZD2
Mmu FZD2
Gga FZD2
Dre FZD2
Tni FZD2
Gga FZD1
Hsa FZD1
Mmu FZD1
Tni FZD7 b
Dre FZD7
Tni FZD7 a
Gga FZD7
Hsa FZD7
Mmu FZD7
Cte 202176
Lgi 139359
Nve 171640
Cin NP 001027713
Nve 183962
Dre fz2-RA
Pdu 94
Cte 140195
Lgi 200873
Dre FZD8c
Tni FZD5
Mmu FZD5
Hsa FZD5
Hsa FZD8
Mmu FZD8
Dre FZD8b
Dre FZD8a
Tni FZD8
Cin FDZ Q4H315
Dme fz-RA
Cin ENSCINT00000005012
Nve 168924
Hsa FZD4
Mmu FZD4
Gga FZD4
Dre FZD4
Pdu 91
Cte 166935
Lgi 201339
Nve 139208
Cte 176783
Lgi 228923
Pdu 117
Hsa FZD9
Mmu FZD9
Dre FZD9 571586
Dre FZD9 A3QK10
Hsa FZD10
Gga FZD10
Dre FZD10
Tni FZD10
Cin FDZ Q4H3G0
Hsa FZD6
Mmu FZD6
Gga FZD6
Tni FZD6
Dre FDZ3
Tni FDZ3
Gga FZD3
Hsa FZD3
Mmu FZD3
Dme FBtr0070138
Cte 108217
Lgi 195892
0.1

# Frizzled ML

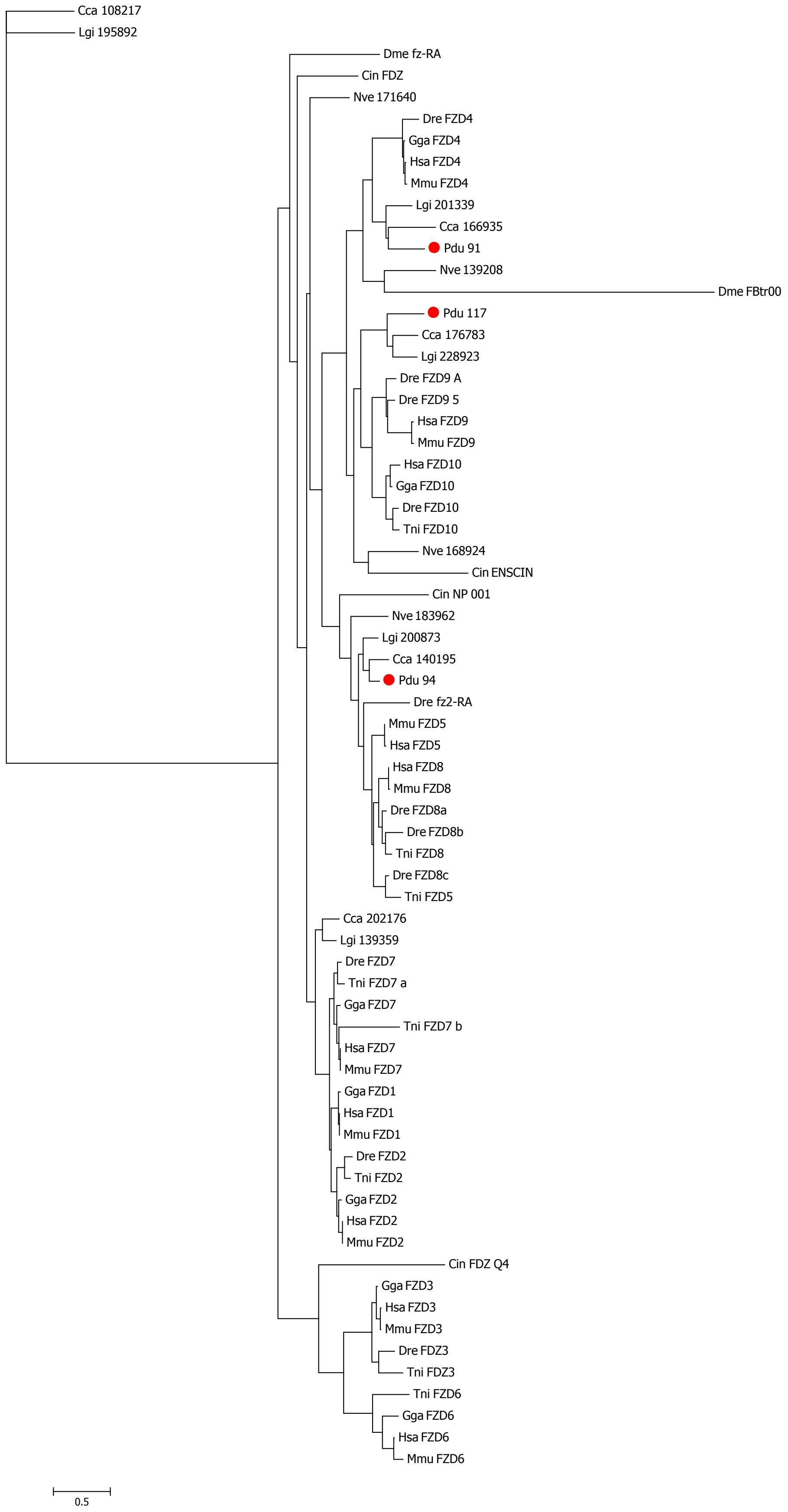

Cca 108217
Lgi 195892
Dme fz-RA
Cin FDZ
Nve 171640
Dre FZD4
Gga FZD4
Hsa FZD4
Mmu FZD4
Lgi 201339
Cca 166935
Pdu 91
Nve 139208
Dme FBtr00
Pdu 117
Cca 176783
Lgi 228923
Dre FZD9 A
Dre FZD9 5
Hsa FZD9
Mmu FZD9
Hsa FZD10
Gga FZD10
Dre FZD10
Tni FZD10
Nve 168924
Cin ENSCIN
Cin NP 001
Nve 183962
Lgi 200873
Cca 140195
Pdu 94
Dre fz2-RA
Mmu FZD5
Hsa FZD5
Hsa FZD8
Mmu FZD8
Dre FZD8a
Dre FZD8b
Tni FZD8
Dre FZD8c
Tni FZD5
Cca 202176
Lgi 139359
Dre FZD7
Tni FZD7 a
Gga FZD7
Tni FZD7 b
Hsa FZD7
Mmu FZD7
Gga FZD1
Hsa FZD1
Mmu FZD1
Dre FZD2
Tni FZD2
Gga FZD2
Hsa FZD2
Mmu FZD2
Cin FDZ Q4
Gga FZD3
Hsa FZD3
Mmu FZD3
Dre FDZ3
Tni FDZ3
Tni FZD6
Gga FZD6
Hsa FZD6
Mmu FZD6
0.5

Gbrl1 NJ

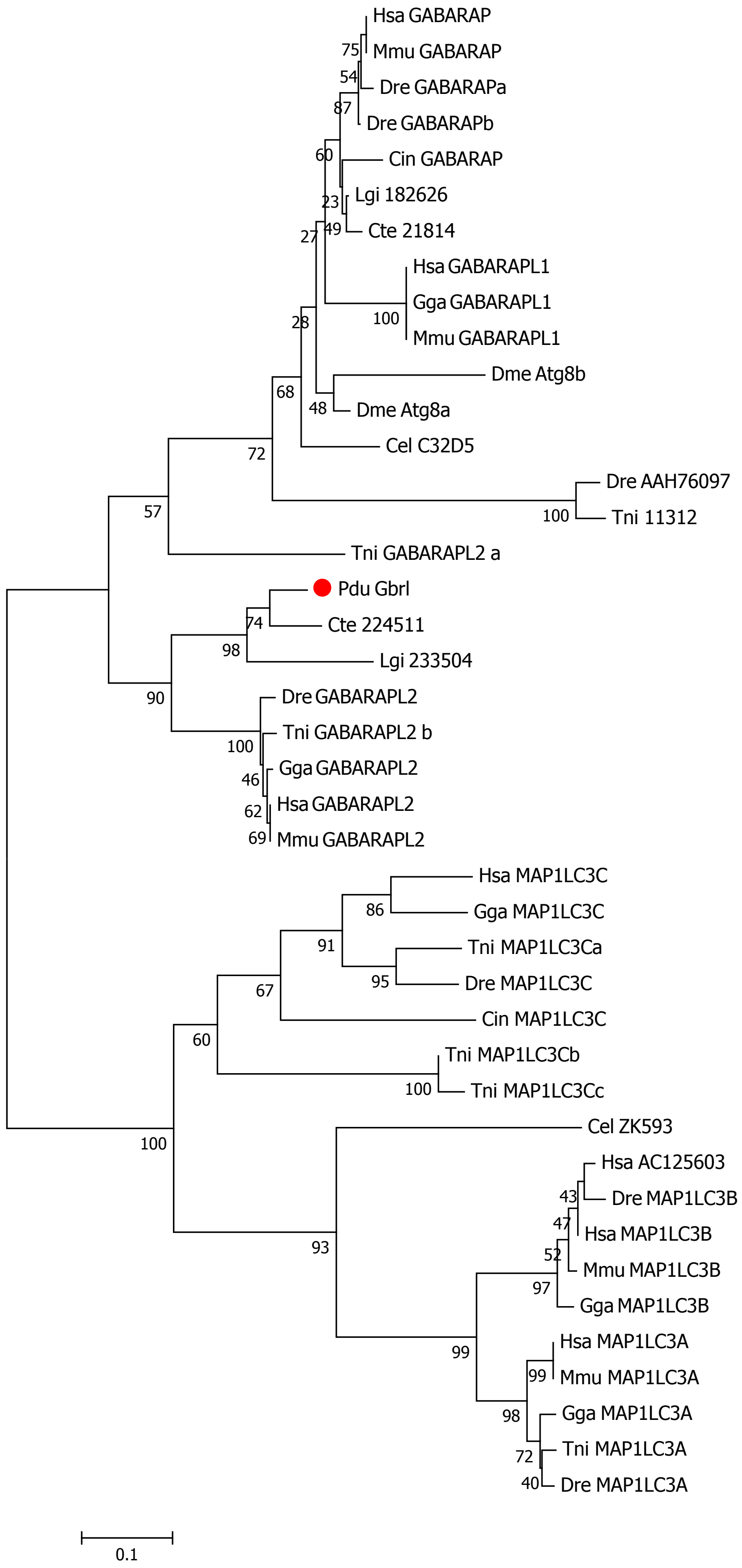

Hsa GABARAP
Mmu GABARAP
Dre GABARAPa
Dre GABARAPb
Cin GABARAP
Lgi 182626
Cte 21814
Hsa GABARAPL1
Gga GABARAPL1
Mmu GABARAPL1
Dme Atg8b
Dme Atg8a
Cel C32D5
Dre AAH76097
Tni 11312
Tni GABARAPL2 a
Pdu Gbrl
Cte 224511
Lgi 233504
Dre GABARAPL2
Tni GABARAPL2 b
Gga GABARAPL2
Hsa GABARAPL2
Mmu GABARAPL2
Hsa MAP1LC3C
Gga MAP1LC3C
Tni MAP1LC3Ca
Dre MAP1LC3C
Cin MAP1LC3C
Tni MAP1LC3Cb
Tni MAP1LC3Cc
Cel ZK593
Hsa AC125603
Dre MAP1LC3B
Hsa MAP1LC3B
Mmu MAP1LC3B
Gga MAP1LC3B
Hsa MAP1LC3A
Mmu MAP1LC3A
Gga MAP1LC3A
Tni MAP1LC3A
Dre MAP1LC3A
0.1

Gbrl ML

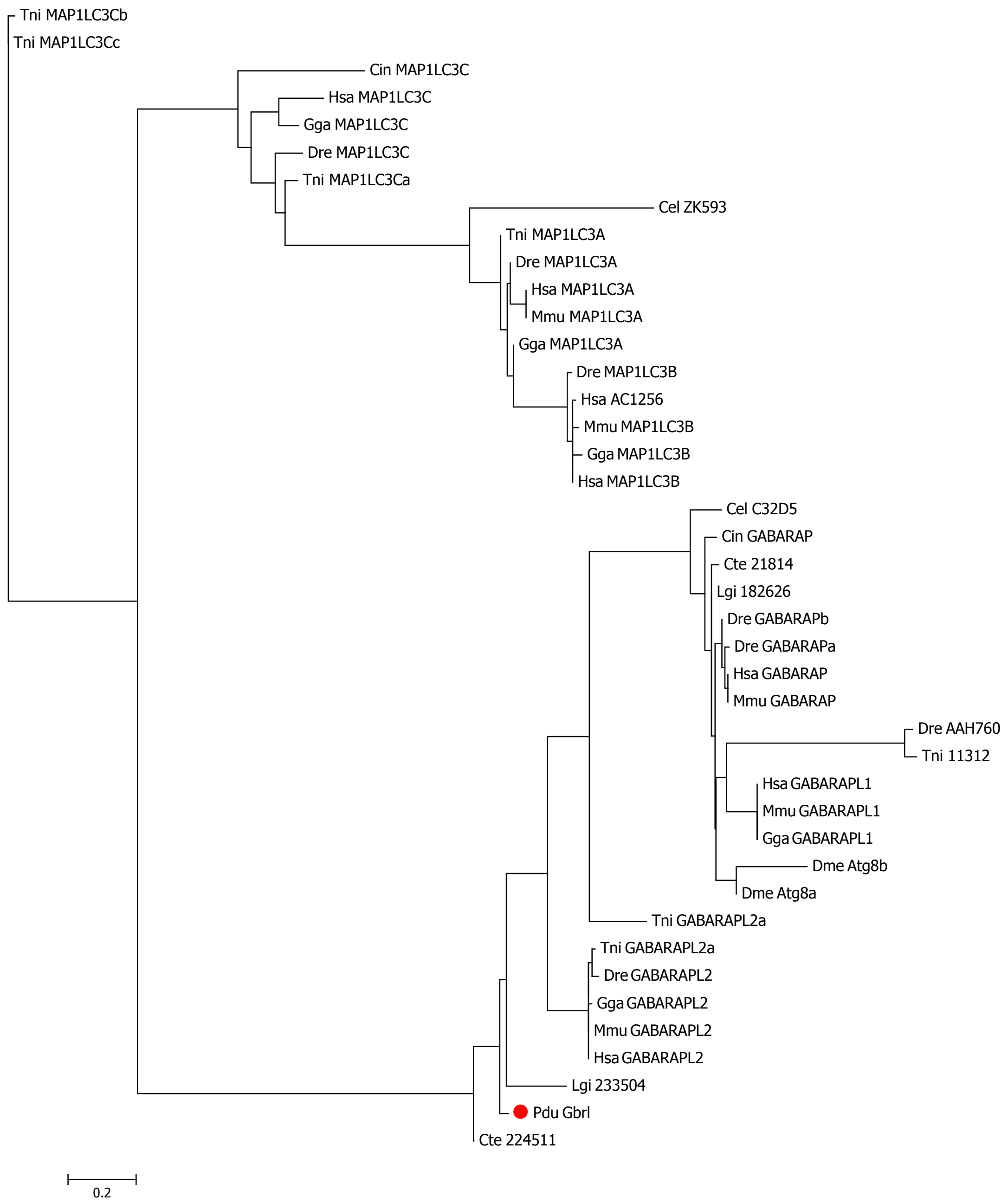
Tni MAP1LC3Cb
Tni MAP1LC3Cc
Cin MAP1LC3C
Hsa MAP1LC3C
Gga MAP1LC3C
Dre MAP1LC3C
Tni MAP1LC3Ca
Cel ZK593
Tni MAP1LC3A
Dre MAP1LC3A
Hsa MAP1LC3A
Mmu MAP1LC3A
Gga MAP1LC3A
Dre MAP1LC3B
Hsa AC1256
Mmu MAP1LC3B
Gga MAP1LC3B
Hsa MAP1LC3B
Cel C32D5
Cin GABARAP
Cte 21814
Lgi 182626
Dre GABARAPb
Dre GABARAPa
Hsa GABARAP
Mmu GABARAP
Dre AAH760
Tni 11312
Hsa GABARAPL1
Mmu GABARAPL1
Gga GABARAPL1
Dme Atg8b
Dme Atg8a
Tni GABARAPL2a
Tni GABARAPL2a
Dre GABARAPL2
Gga GABARAPL2
Mmu GABARAPL2
Hsa GABARAPL2
Lgi 233504
Pdu Gbrl
Cte 224511
0.2

# Klf NJ

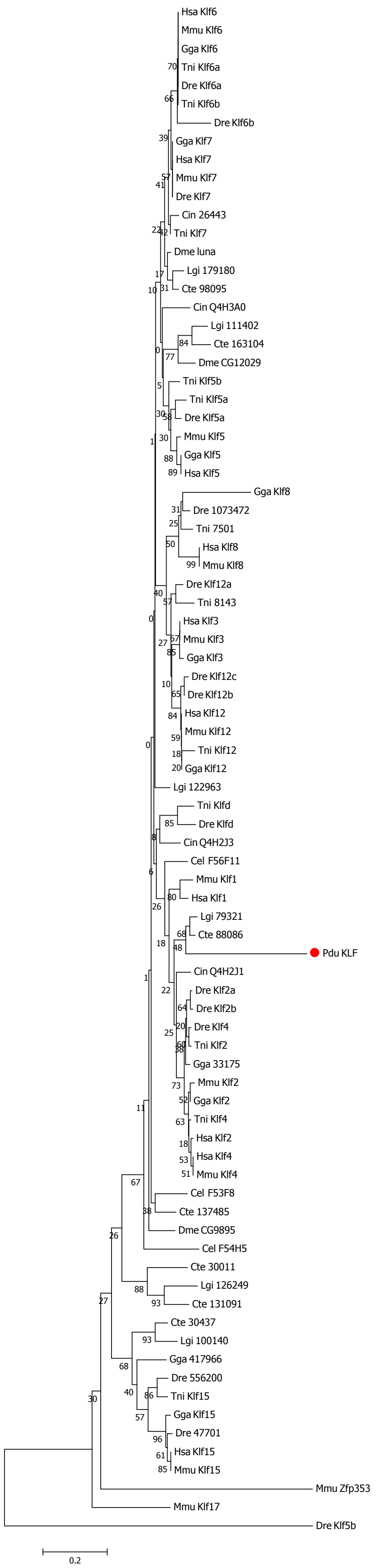

Hsa Klf6
Mmu Klf6
Gga Klf6
Tni Klf6a
Dre Klf6a
Tni Klf6b
Dre Klf6b
Gga Klf7
Hsa Klf7
Mmu Klf7
Dre Klf7
Cin 26443
Tni Klf7
Dme luna
Lgi 179180
Cte 98095
Cin Q4H3A0
Lgi 111402
Cte 163104
Dme CG12029
Tni Klf5b
Tni Klf5a
Dre Klf5a
Mmu Klf5
Gga Klf5
Hsa Klf5
Gga Klf8
Dre 1073472
Tni 7501
Hsa Klf8
Mmu Klf8
Dre Klf12a
Tni 8143
Hsa Klf3
Mmu Klf3
Gga Klf3
Dre Klf12c
Dre Klf12b
Hsa Klf12
Mmu Klf12
Tni Klf12
Gga Klf12
Lgi 122963
Tni Klfd
Dre Klfd
Cin Q4H2J3
Cel F56F11
Mmu Klf1
Hsa Klf1
Lgi 79321
Cte 88086
Pdu KLF
Cin Q4H2J1
Dre Klf2a
Dre Klf2b
Dre Klf4
Tni Klf2
Gga 33175
Mmu Klf2
Gga Klf2
Tni Klf4
Hsa Klf2
Hsa Klf4
Mmu Klf4
Cel F53F8
Cte 137485
Dme CG9895
Cel F54H5
Cte 30011
Lgi 126249
Cte 131091
Cte 30437
Lgi 100140
Gga 417966
Dre 556200
Tni Klf15
Gga Klf15
Dre 47701
Hsa Klf15
Mmu Klf15
Mmu Zfp353
Mmu Klf17
Dre Klf5b
0.2

# Klf ML

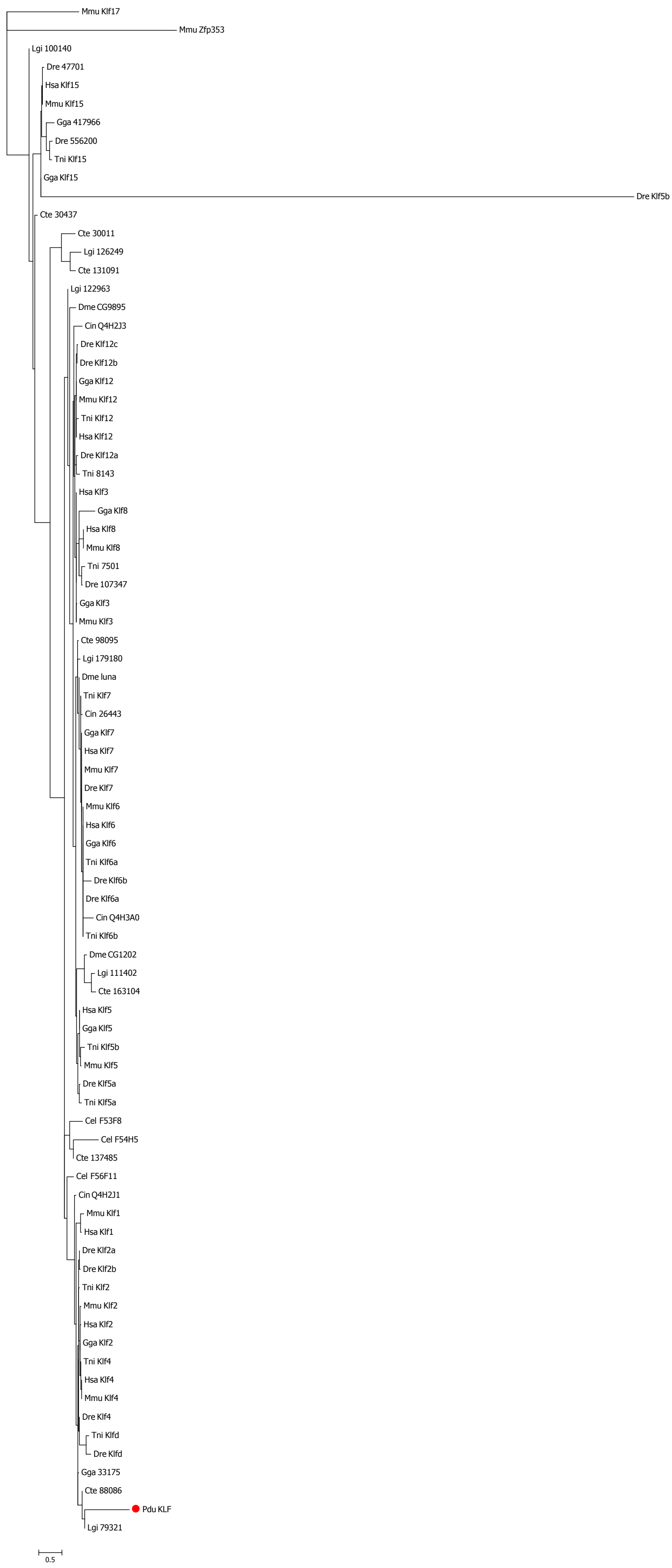

Mmu Klf17
Mmu Zfp353
Lgi 100140
Dre 47701
Hsa Klf15
Mmu Klf15
Gga 417966
Dre 556200
Tni Klf15
Gga Klf15
Dre Klf5b
Cte 30437
Cte 30011
Lgi 126249
Cte 131091
Lgi 122963
Dme CG9895
Cin Q4H2J3
Dre Klf12c
Dre Klf12b
Gga Klf12
Mmu Klf12
Tni Klf12
Hsa Klf12
Dre Klf12a
Tni 8143
Hsa Klf3
Gga Klf8
Hsa Klf8
Mmu Klf8
Tni 7501
Dre 107347
Gga Klf3
Mmu Klf3
Cte 98095
Lgi 179180
Dme luna
Tni Klf7
Cin 26443
Gga Klf7
Hsa Klf7
Mmu Klf7
Dre Klf7
Mmu Klf6
Hsa Klf6
Gga Klf6
Tni Klf6a
Dre Klf6b
Dre Klf6a
Cin Q4H3A0
Tni Klf6b
Dme CG1202
Lgi 111402
Cte 163104
Hsa Klf5
Gga Klf5
Tni Klf5b
Mmu Klf5
Dre Klf5a
Tni Klf5a
Cel F53F8
Cel F54H5
Cte 137485
Cel F56F11
Cin Q4H2J1
Mmu Klf1
Hsa Klf1
Dre Klf2a
Dre Klf2b
Tni Klf2
Mmu Klf2
Hsa Klf2
Gga Klf2
Tni Klf4
Hsa Klf4
Mmu Klf4
Dre Klf4
Tni Klfd
Dre Klfd
Gga 33175
Cte 88086
Pdu KLF
Lgi 79321
0.5

Onecut NJ

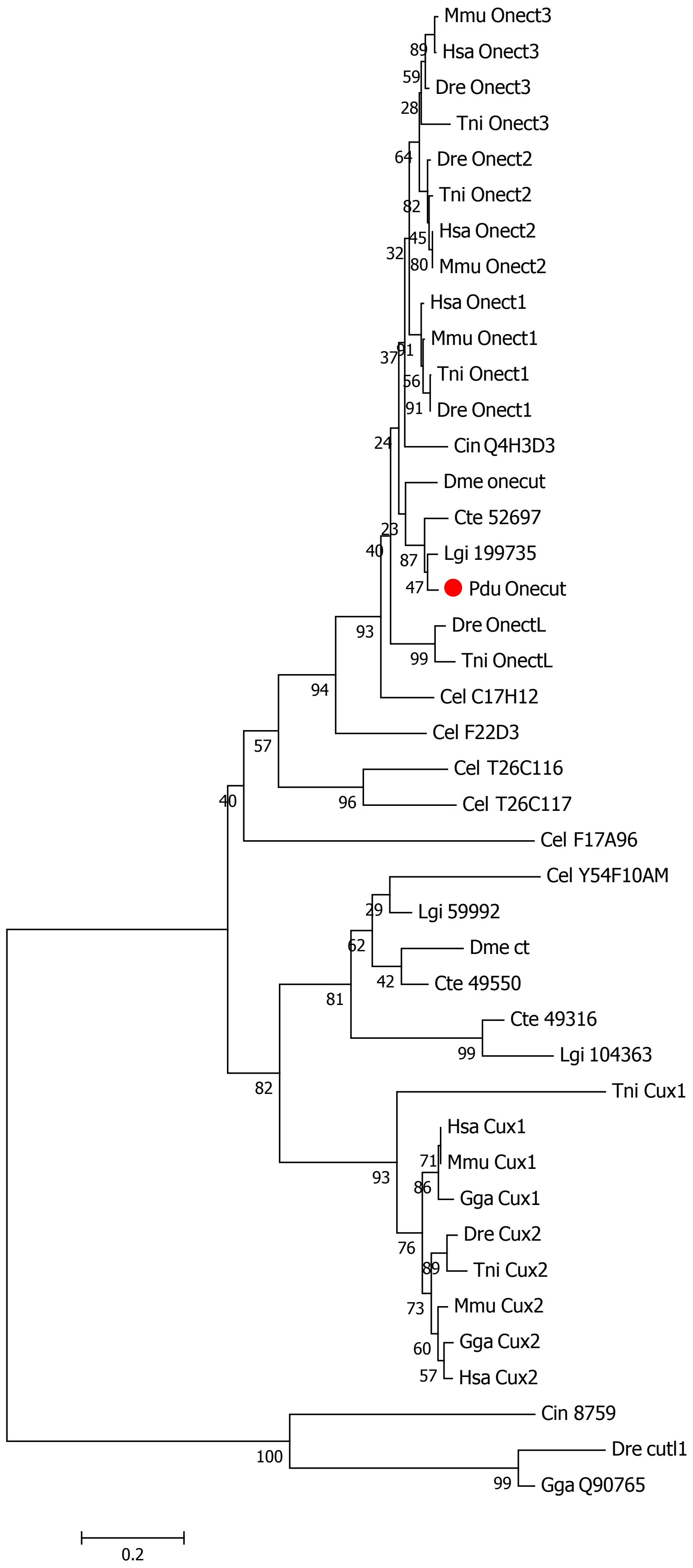

Mmu Onect3
Hsa Onect3
Dre Onect3
Tni Onect3
Dre Onect2
Tni Onect2
Hsa Onect2
Mmu Onect2
Hsa Onect1
Mmu Onect1
Tni Onect1
Dre Onect1
Cin Q4H3D3
Dme onecut
Cte 52697
Lgi 199735
Pdu Onecut
Dre OnectL
Tni OnectL
Cel C17H12
Cel F22D3
Cel T26C116
Cel T26C117
Cel F17A96
Cel Y54F10AM
Lgi 59992
Dme ct
Cte 49550
Cte 49316
Lgi 104363
Tni Cux1
Hsa Cux1
Mmu Cux1
Gga Cux1
Dre Cux2
Tni Cux2
Mmu Cux2
Gga Cux2
Hsa Cux2
Cin 8759
Dre cutl1
Gga Q90765
89
59
28
64
82
45
32
80
37
91
56
91
24
23
40
87
47
93
99
94
57
96
40
29
62
42
81
99
82
71
86
93
76
89
73
60
57
100
99
0.2

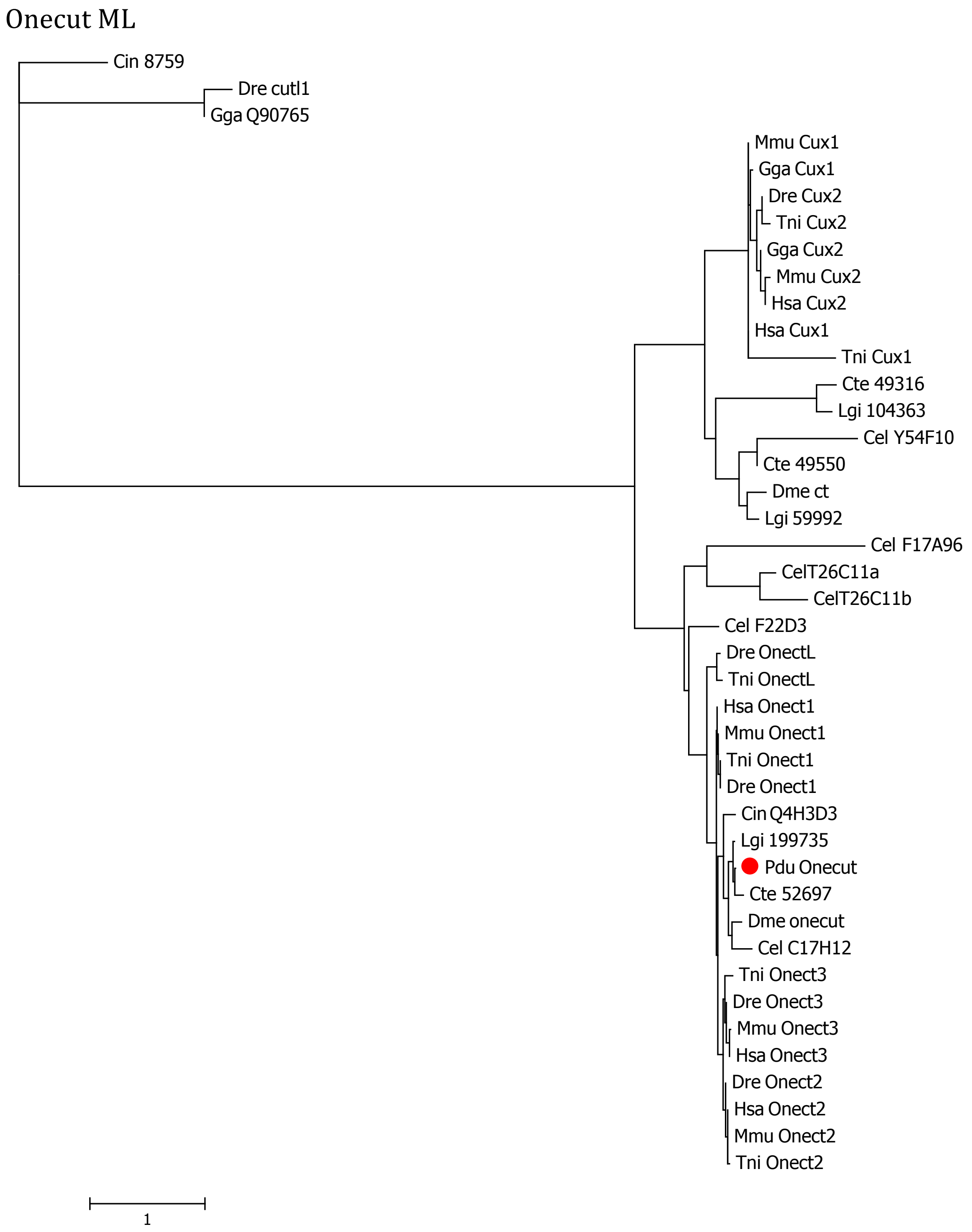

Onecut ML
Cin 8759
Dre cutl1
Gga Q90765
Mmu Cux1
Gga Cux1
Dre Cux2
Tni Cux2
Gga Cux2
Mmu Cux2
Hsa Cux2
Hsa Cux1
Tni Cux1
Cte 49316
Lgi 104363
Cel Y54F10
Cte 49550
Dme ct
Lgi 59992
Cel F17A96
CelT26C11a
CelT26C11b
Cel F22D3
Dre OnectL
Tni OnectL
Hsa Onect1
Mmu Onect1
Tni Onect1
Dre Onect1
Cin Q4H3D3
Lgi 199735
Pdu Onecut
Cte 52697
Dme onecut
Cel C17H12
Tni Onect3
Dre Onect3
Mmu Onect3
Hsa Onect3
Dre Onect2
Hsa Onect2
Mmu Onect2
Tni Onect2
1

## Sfrp NJ

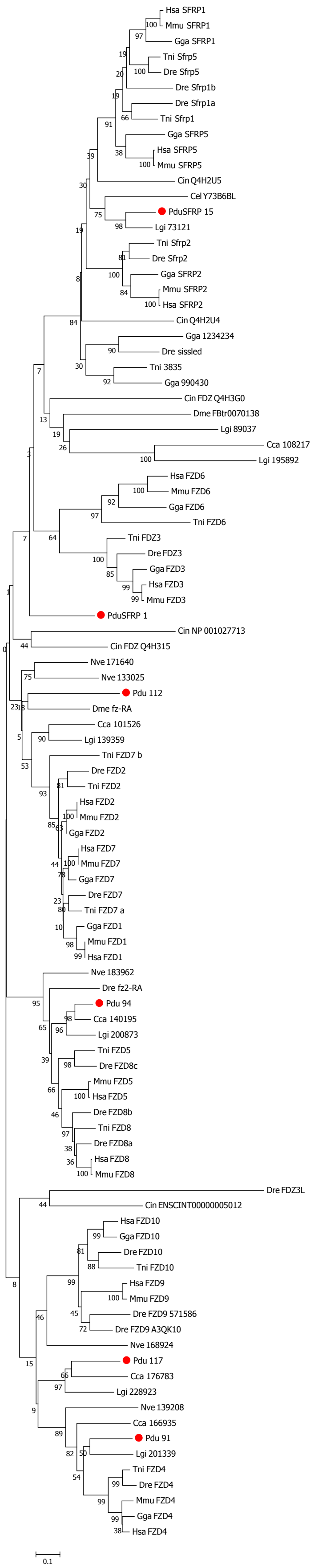

Hsa SFRP1
Mmu SFRP1
Gga SFRP1
Tni Sfrp5
Dre Sfrp5
Dre Sfrp1b
Dre Sfrp1a
Tni Sfrp1
Gga SFRP5
Hsa SFRP5
Mmu SFRP5
Cin Q4H2U5
Cel Y73B6BL
PduSFRP 15
Lgi 73121
Tni Sfrp2
Dre Sfrp2
Gga SFRP2
Mmu SFRP2
Hsa SFRP2
Cin Q4H2U4
Gga 1234234
Dre sissled
Tni 3835
Gga 990430
Cin FDZ Q4H3G0
Dme FBtr0070138
Lgi 89037
Cca 108217
Lgi 195892
Hsa FZD6
Mmu FZD6
Gga FZD6
Tni FZD6
Tni FDZ3
Dre FDZ3
Gga FZD3
Hsa FZD3
Mmu FZD3
PduSFRP 1
Cin NP 001027713
Cin FDZ Q4H315
Nve 171640
Nve 133025
Pdu 112
Dme fz-RA
Cca 101526
Lgi 139359
Tni FZD7 b
Dre FZD2
Tni FZD2
Hsa FZD2
Mmu FZD2
Gga FZD2
Hsa FZD7
Mmu FZD7
Gga FZD7
Dre FZD7
Tni FZD7 a
Gga FZD1
Mmu FZD1
Hsa FZD1
Nve 183962
Dre fz2-RA
Pdu 94
Cca 140195
Lgi 200873
Tni FZD5
Dre FZD8c
Mmu FZD5
Hsa FZD5
Dre FZD8b
Tni FZD8
Dre FZD8a
Hsa FZD8
Mmu FZD8
Dre FDZ3L
Cin ENSCINT00000005012
Hsa FZD10
Gga FZD10
Dre FZD10
Tni FZD10
Hsa FZD9
Mmu FZD9
Dre FZD9 571586
Dre FZD9 A3QK10
Nve 168924
Pdu 117
Cca 176783
Lgi 228923
Nve 139208
Cca 166935
Pdu 91
Lgi 201339
Tni FZD4
Dre FZD4
Mmu FZD4
Gga FZD4
Hsa FZD4
0.1

# Sfrp ML

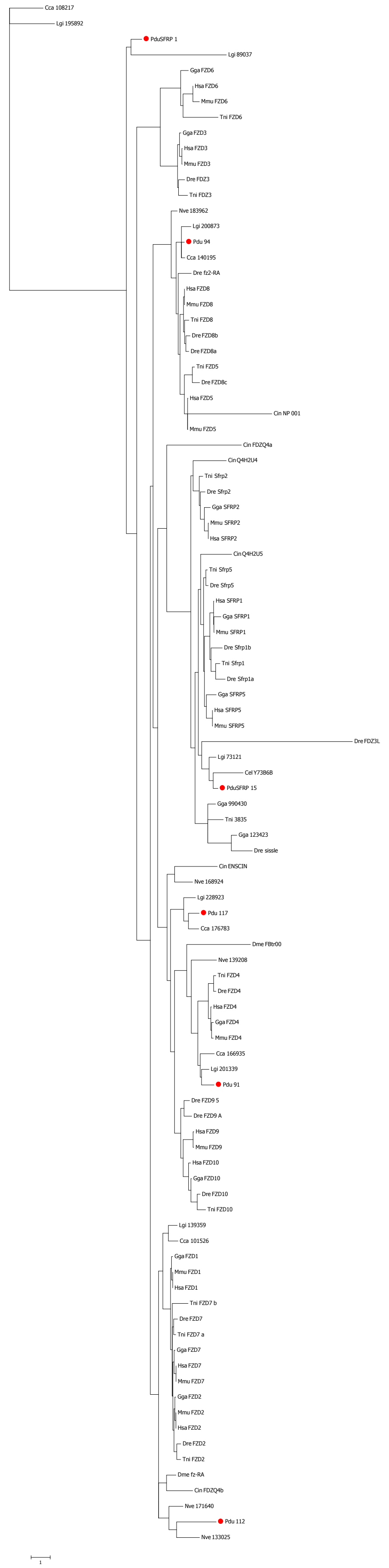
Cca 108217
Lgi 195892
PduSFRP 1
Lgi 89037
Gga FZD6
Hsa FZD6
Mmu FZD6
Tni FZD6
Gga FZD3
Hsa FZD3
Mmu FZD3
Dre FDZ3
Tni FDZ3
Nve 183962
Lgi 200873
Pdu 94
Cca 140195
Dre fz2-RA
Hsa FZD8
Mmu FZD8
Tni FZD8
Dre FZD8b
Dre FZD8a
Tni FZD5
Dre FZD8c
Hsa FZD5
Cin NP 001
Mmu FZD5
Cin FDZQ4a
Cin Q4H2U4
Tni Sfrp2
Dre Sfrp2
Gga SFRP2
Mmu SFRP2
Hsa SFRP2
Cin Q4H2U5
Tni Sfrp5
Dre Sfrp5
Hsa SFRP1
Gga SFRP1
Mmu SFRP1
Dre Sfrp1b
Tni Sfrp1
Dre Sfrp1a
Gga SFRP5
Hsa SFRP5
Mmu SFRP5
Dre FDZ3L
Lgi 73121
Cel Y73B6B
PduSFRP 15
Gga 990430
Tni 3835
Gga 123423
Dre sissle
Cin ENSCIN
Nve 168924
Lgi 228923
Pdu 117
Cca 176783
Dme FBtr00
Nve 139208
Tni FZD4
Dre FZD4
Hsa FZD4
Gga FZD4
Mmu FZD4
Cca 166935
Lgi 201339
Pdu 91
Dre FZD9 5
Dre FZD9 A
Hsa FZD9
Mmu FZD9
Hsa FZD10
Gga FZD10
Dre FZD10
Tni FZD10
Lgi 139359
Cca 101526
Gga FZD1
Mmu FZD1
Hsa FZD1
Tni FZD7 b
Dre FZD7
Tni FZD7 a
Gga FZD7
Hsa FZD7
Mmu FZD7
Gga FZD2
Mmu FZD2
Hsa FZD2
Dre FZD2
Tni FZD2
Dme fz-RA
Cin FDZQ4b
Nve 171640
Pdu 112
Nve 133025
1

Spondin NJ

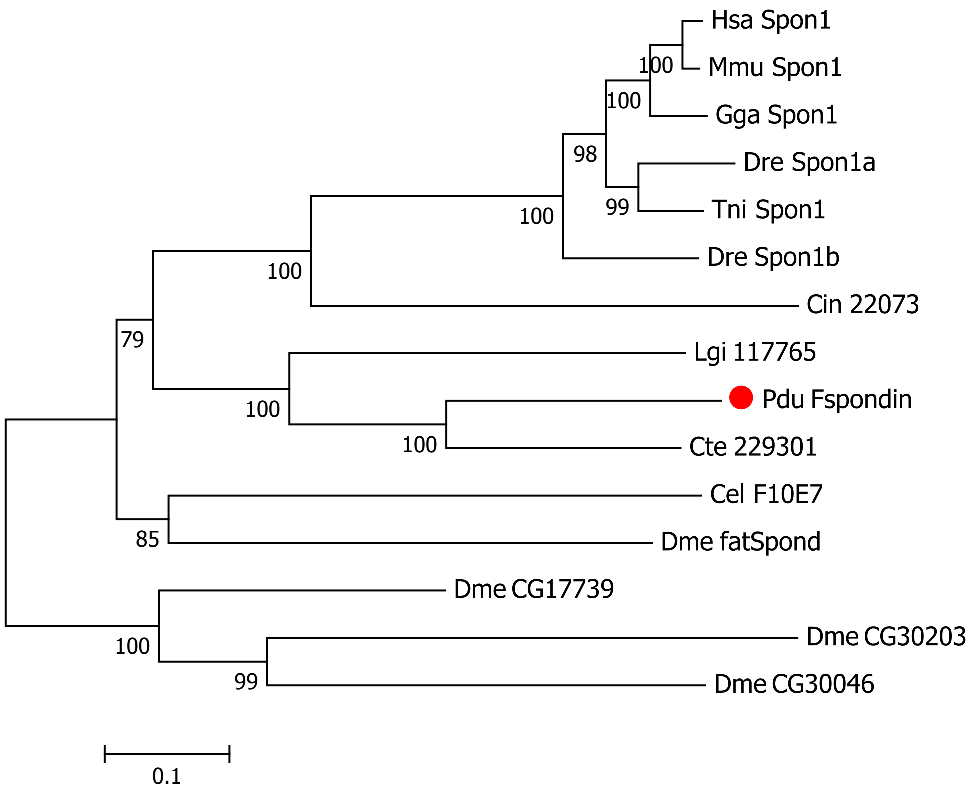


Spondin ML

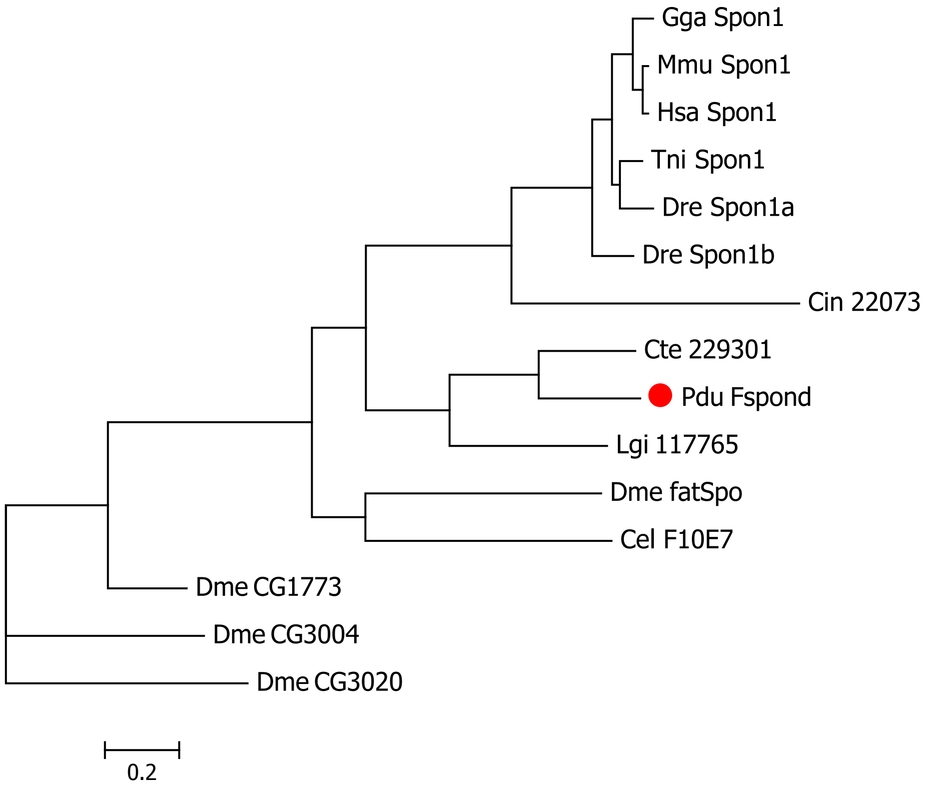

Tektin2 NJ

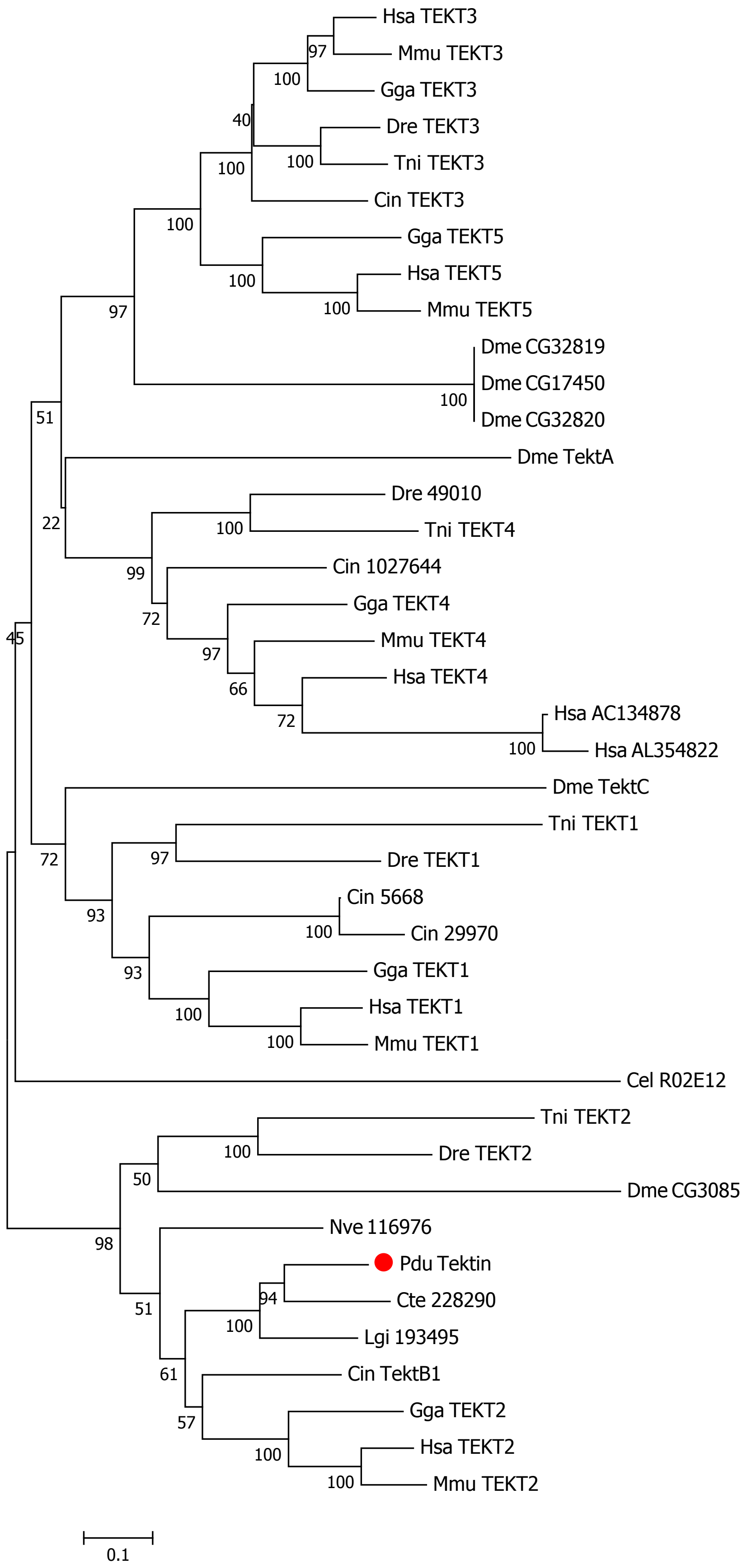
Hsa TEKT3
Mmu TEKT3
Gga TEKT3
Dre TEKT3
Tni TEKT3
Cin TEKT3
Gga TEKT5
Hsa TEKT5
Mmu TEKT5
Dme CG32819
Dme CG17450
Dme CG32820
Dme TektA
Dre 49010
Tni TEKT4
Cin 1027644
Gga TEKT4
Mmu TEKT4
Hsa TEKT4
Hsa AC134878
Hsa AL354822
Dme TektC
Tni TEKT1
Dre TEKT1
Cin 5668
Cin 29970
Gga TEKT1
Hsa TEKT1
Mmu TEKT1
Cel R02E12
Tni TEKT2
Dre TEKT2
Dme CG3085
Nve 116976
Pdu Tektin
Cte 228290
Lgi 193495
Cin TektB1
Gga TEKT2
Hsa TEKT2
Mmu TEKT2
97
100
40
100
100
100
100
100
97
100
51
22
100
99
72
97
66
72
100
45
97
72
93
100
93
100
100
100
50
98
94
100
51
61
57
100
100
0.1

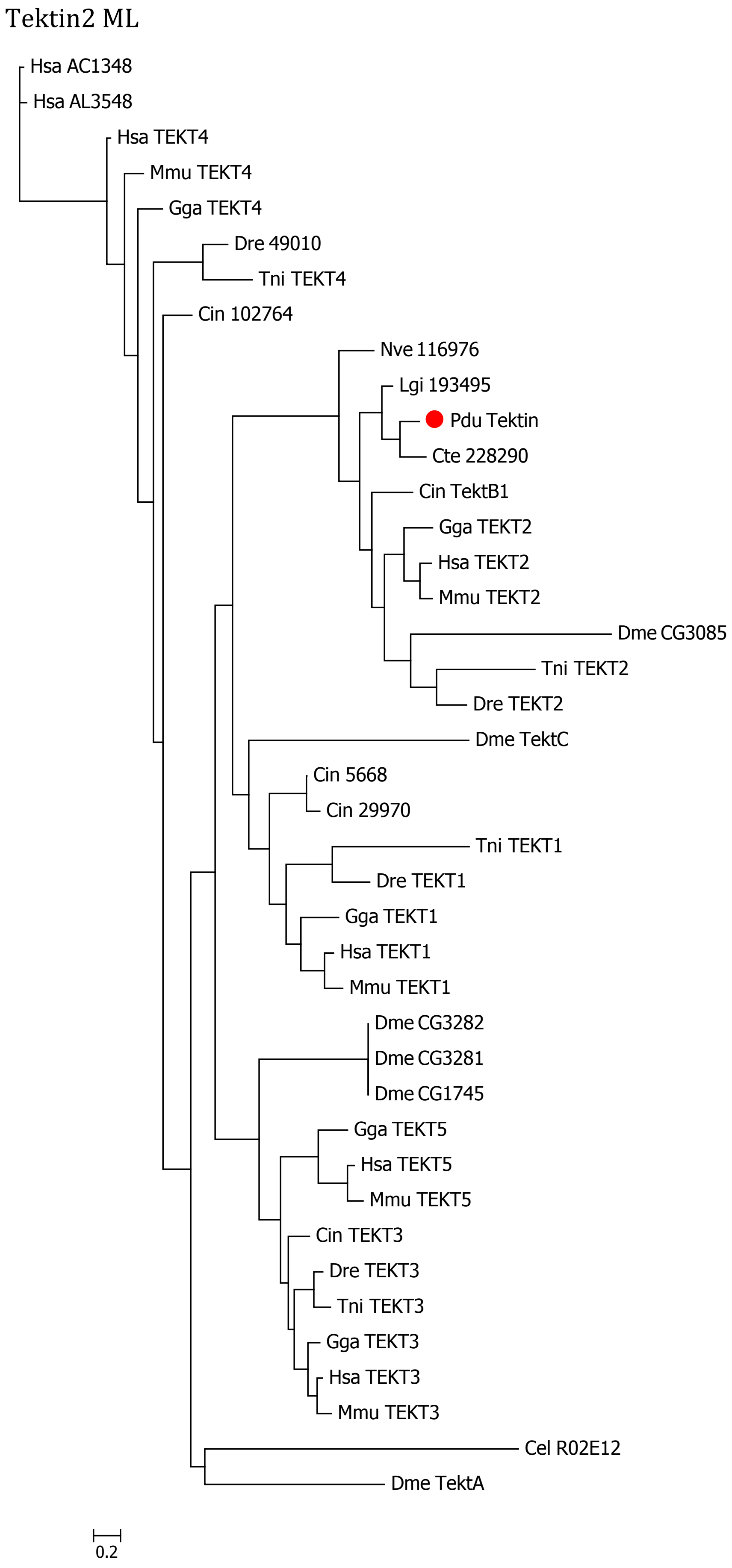
Tektin2 ML
Hsa AC1348
Hsa AL3548
Hsa TEKT4
Mmu TEKT4
Gga TEKT4
Dre 49010
Tni TEKT4
Cin 102764
Nve 116976
Lgi 193495
Pdu Tektin
Cte 228290
Cin TektB1
Gga TEKT2
Hsa TEKT2
Mmu TEKT2
Dme CG3085
Tni TEKT2
Dre TEKT2
Dme TektC
Cin 5668
Cin 29970
Tni TEKT1
Dre TEKT1
Gga TEKT1
Hsa TEKT1
Mmu TEKT1
Dme CG3282
Dme CG3281
Dme CG1745
Gga TEKT5
Hsa TEKT5
Mmu TEKT5
Cin TEKT3
Dre TEKT3
Tni TEKT3
Gga TEKT3
Hsa TEKT3
Mmu TEKT3
Cel R02E12
Dme TektA
0.2

Tubby NJ

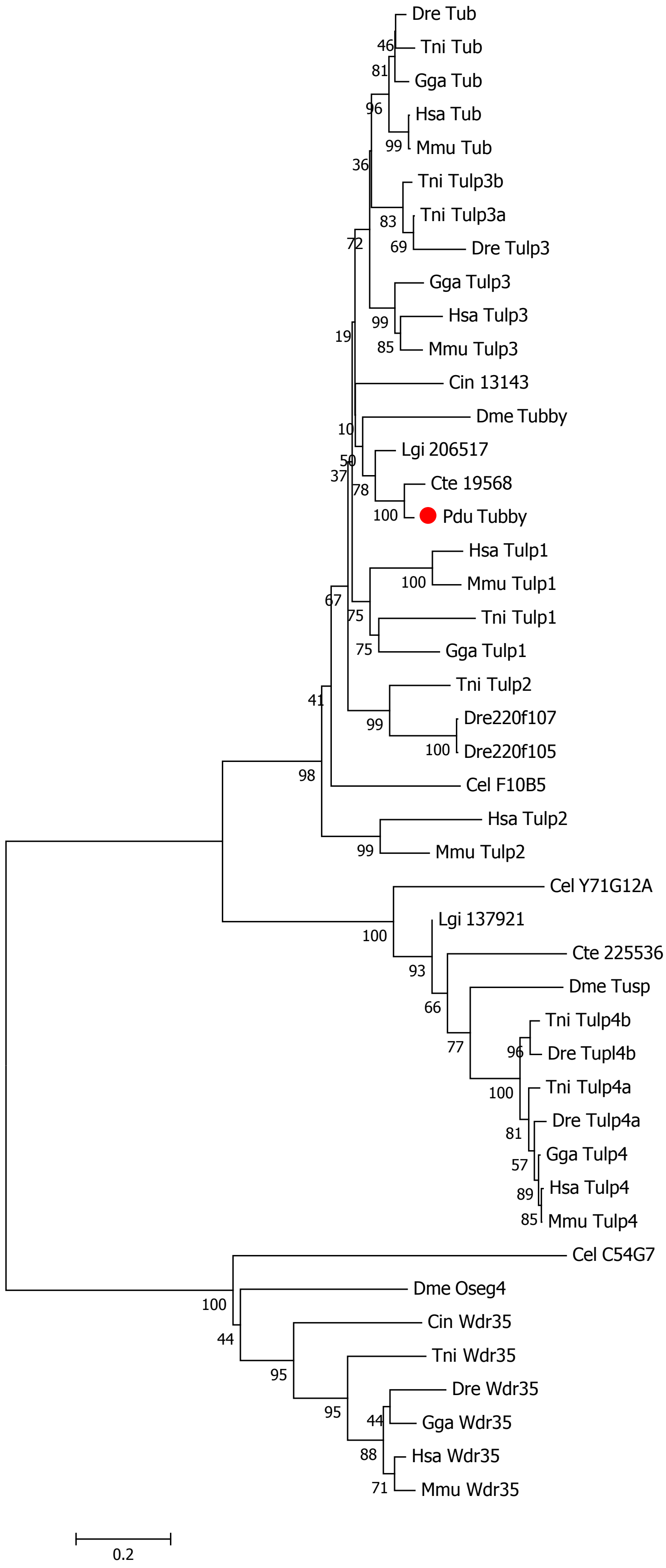
Dre Tub
Tni Tub
Gga Tub
Hsa Tub
Mmu Tub
Tni Tulp3b
Tni Tulp3a
Dre Tulp3
Gga Tulp3
Hsa Tulp3
Mmu Tulp3
Cin 13143
Dme Tubby
Lgi 206517
Cte 19568
Pdu Tubby
Hsa Tulp1
Mmu Tulp1
Tni Tulp1
Gga Tulp1
Tni Tulp2
Dre220f107
Dre220f105
Cel F10B5
Hsa Tulp2
Mmu Tulp2
Cel Y71G12A
Lgi 137921
Cte 225536
Dme Tusp
Tni Tulp4b
Dre Tupl4b
Tni Tulp4a
Dre Tulp4a
Gga Tulp4
Hsa Tulp4
Mmu Tulp4
Cel C54G7
Dme Oseg4
Cin Wdr35
Tni Wdr35
Dre Wdr35
Gga Wdr35
Hsa Wdr35
Mmu Wdr35
0.2

# Tubby ML

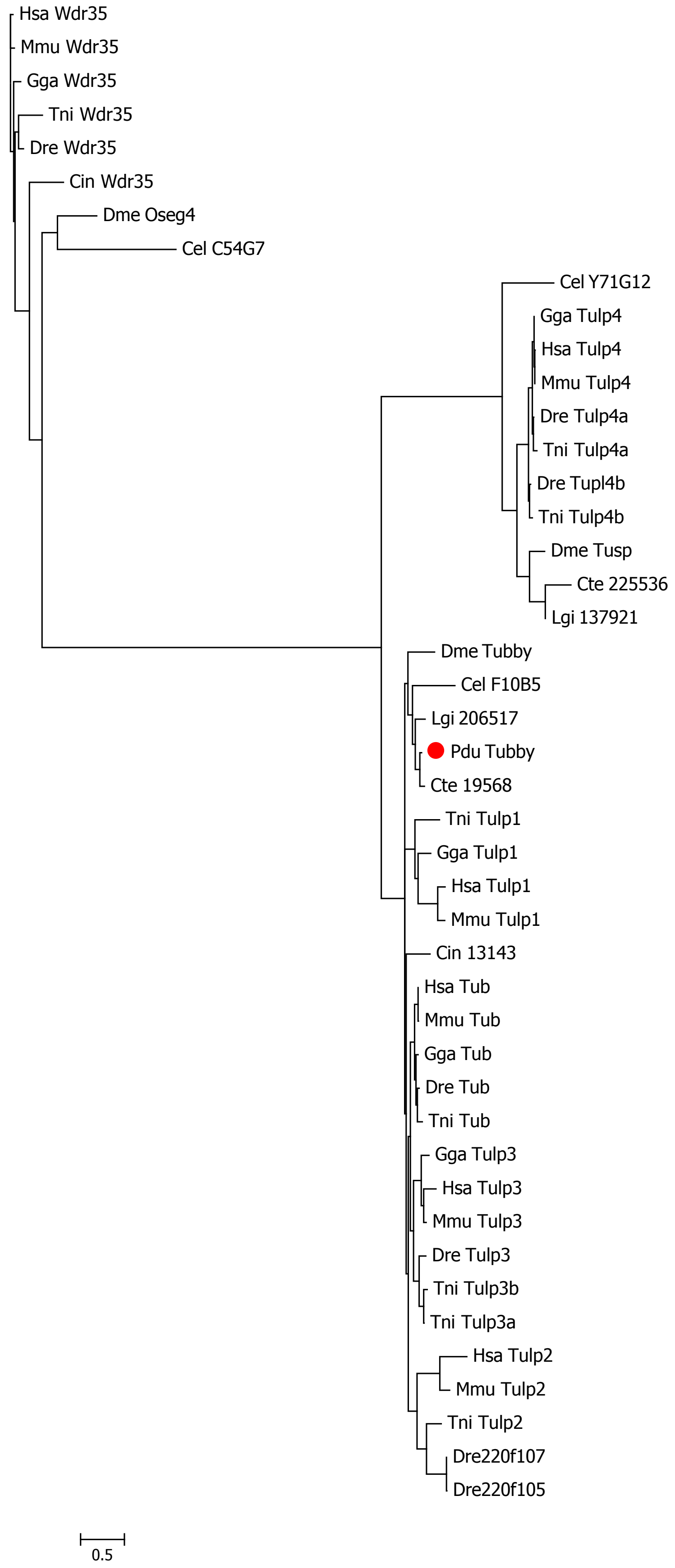
Hsa Wdr35
Mmu Wdr35
Gga Wdr35
Tni Wdr35
Dre Wdr35
Cin Wdr35
Dme Oseg4
Cel C54G7
Cel Y71G12
Gga Tulp4
Hsa Tulp4
Mmu Tulp4
Dre Tulp4a
Tni Tulp4a
Dre Tupl4b
Tni Tulp4b
Dme Tusp
Cte 225536
Lgi 137921
Dme Tubby
Cel F10B5
Lgi 206517
Pdu Tubby
Cte 19568
Tni Tulp1
Gga Tulp1
Hsa Tulp1
Mmu Tulp1
Cin 13143
Hsa Tub
Mmu Tub
Gga Tub
Dre Tub
Tni Tub
Gga Tulp3
Hsa Tulp3
Mmu Tulp3
Dre Tulp3
Tni Tulp3b
Tni Tulp3a
Hsa Tulp2
Mmu Tulp2
Tni Tulp2
Dre220f107
Dre220f105
0.5
